# Molecular details of dimerization kinetics reveal negligible populations of transient µ-opioid receptor homodimers at physiological concentrations

**DOI:** 10.1038/s41598-018-26070-8

**Published:** 2018-05-16

**Authors:** Derya Meral, Davide Provasi, Diego Prada-Gracia, Jan Möller, Kristen Marino, Martin J. Lohse, Marta Filizola

**Affiliations:** 1 Department of Pharmacological Sciences, Icahn School of Medicine at Mount Sinai, New York, NY USA; 2 Max Delbrück Center for Molecular Medicine, Berlin, Germany; 3 Institute of Pharmacology and Toxicology, Würzburg, Germany

## Abstract

Various experimental and computational techniques have been employed over the past decade to provide structural and thermodynamic insights into G Protein-Coupled Receptor (GPCR) dimerization. Here, we use multiple microsecond-long, coarse-grained, biased and unbiased molecular dynamics simulations (a total of ~4 milliseconds) combined with multi-ensemble Markov state models to elucidate the kinetics of homodimerization of a prototypic GPCR, the µ-opioid receptor (MOR), embedded in a 1-palmitoyl-2-oleoyl-sn-glycero-3-phosphocholine (POPC)/cholesterol lipid bilayer. Analysis of these computations identifies kinetically distinct macrostates comprising several different short-lived dimeric configurations of either inactive or activated MOR. Calculated kinetic rates and fractions of dimers at different MOR concentrations suggest a negligible population of MOR homodimers at physiological concentrations, which is supported by acceptor photobleaching fluorescence resonance energy transfer (FRET) experiments. This study provides a rigorous, quantitative explanation for some conflicting experimental data on GPCR oligomerization.

## Introduction

Methodological limitations and inadequate structural information have thus far hampered a full delineation of the thermodynamics, kinetics, and stoichiometry of G Protein-Coupled Receptor (GPCR) interactions in living cells, including the role of di-/oligomerization in GPCR function. The majority of published studies examining di-/oligomerization report on effects derived from activation of one receptor in the presence of another (e.g., see^1^) or modulation of the activity of one GPCR using ligands targeting another one (e.g., see^2,3^). Although there is clear evidence of signaling from a dimeric unit in the case of GPCR members of the glutamate family^4,5^, differential signaling of a receptor complex is still a matter of debate for rhodopsin family GPCRs^6–10^ in spite of unambiguous evidence of physical interaction for many of them^11,12^, most recently provided by single-molecule imaging^13–15^. Notably, these studies suggest transient formation of receptor dimers, with only a small fraction of the receptors existing as dimers at any given time.

Although very powerful and informative, the experimental technologies employed so far do not provide the level of molecular detail that is required to eventually understand the role of di-/oligomerization in GPCR function. To this end, molecular dynamics (MD) simulations provide particularly useful information, and we and others have used various MD-based techniques to derive structural and thermodynamic information about GPCR dimers/oligomers (e.g.^16,17^). Using the opioid receptors as model systems, we recently carried out extensive MD simulations of coarse-grained (CG) representations of arrays of 16 inactive µ-opioid receptors (MOR), δ-opioid receptors (DOR), or κ-opioid receptors (KOR) to study their self-assembly in a membrane-mimetic environment^16,18^. While umbrella sampling simulations of putative dimer interfaces of inactive MOR or KOR inferred by their crystal structures (i.e., the symmetric interfaces involving transmembrane helices (TM) 5 and 6 (TM5,6/TM5,6), and/or transmembrane helices 1, 2 and helix 8 (TM1,2,H8/TM1,2,H8)) confirmed their thermodynamic stability in a 1-palmitoyl,2-oleoyl-sn-glycero-3-phosphocholine (POPC)/10% cholesterol (CHOL) membrane model, the TM5,6/TM5,6 interface did not form during unbiased simulations of receptor self-association, leading us to conclude that this interface was kinetically unable to form on the microsecond timescale of those simulations^16^.

Here, we take advantage of state-of-the-art algorithms using biased and unbiased MD trajectories to investigate longer timescales, and provide, for the first time, molecular details of the kinetics of the GPCR dimerization process through Markov state model (MSM) analysis of multiple microsecond-long, coarse-grained simulations of pairs of inactive or activated MOR crystal structures embedded in a POPC/CHOL membrane model. The results of this analysis are supported by acceptor photobleaching fluorescence resonance energy transfer (FRET) experiments.

## Results

With the intention of sampling the association/dissociation between inactive or activated structures of MOR, 352 independent systems with randomly-oriented protomer pairs for each receptor conformation were simulated for at least 5 μs each using the MARTINI force field^19–22^, adding up to ~2 milliseconds (ms) of total simulation time for each system (see Supplementary Table 1 for a summary of all simulations presented here). The resulting simulation data were recast in terms of three collective variables (CVs), specifically: (i) the distance *d* between the centers-of-mass (COM) of the protomers, (ii) the angle α_1_ formed between the COM of transmembrane helix 1 of one protomer, its COM, and the COM of the second protomer, and (iii) the angle α_2_ obtained by swapping the two protomers. The resulting distributions of the relative position of the protomers sampled during unbiased simulations of inactive or activated MOR structures can be seen in Supplementary Fig. 1, where the three aforementioned CVs, [*d*, *α*_1_, *α*_2_], have been projected onto two dimensions to obtain the probability distibution of the relative position of the protomers’ centers of mass, i.e. x = *d* cos*α* and *y* = *d* sin*α* (see figure legend for additional details). A preliminary MSM was built after k-means clustering of the CVs to help select regions that are relevant to receptor dimerization using transition path theory (TPT). Configurations within these regions were selected for further exploration by umbrella sampling in order to aid convergence (see Methods section for details). Subsequently, the unbiased and biased simulations were analyzed in tandem using the transition-based reweighting method (TRAM)^23^. In the following sections, we present the structural and kinetic information obtained from the multi-ensemble MSM produced by the TRAM analysis.

### Preferred orientations of inactive or activated MOR structures in putative dimeric configurations

**Figure 1:**
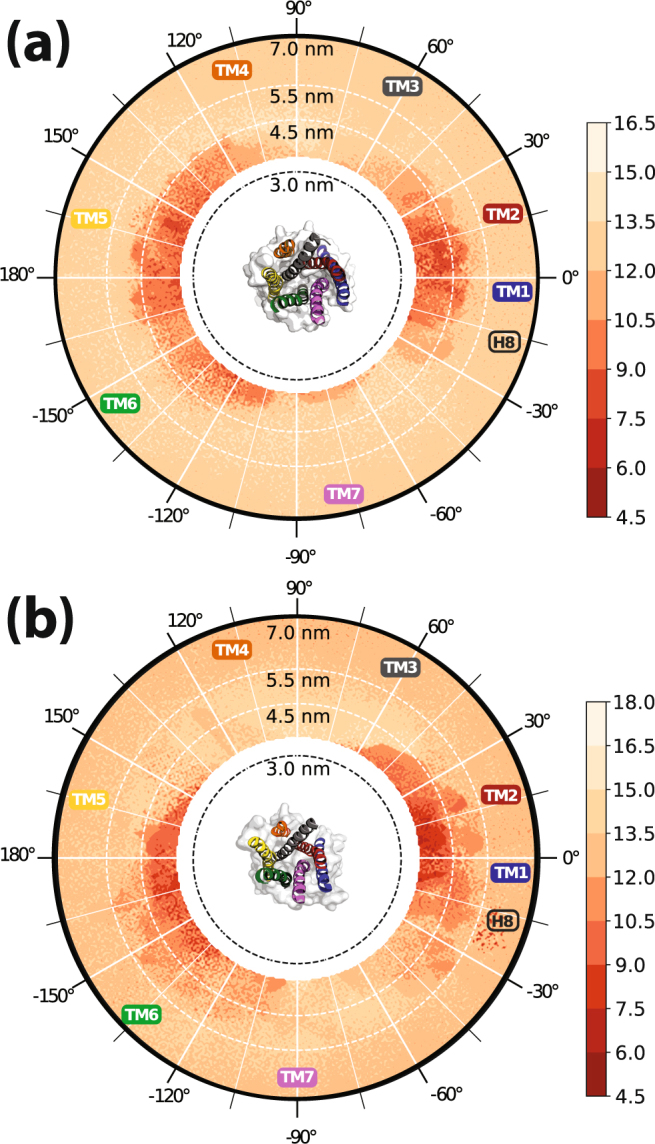
Free energy landscapes of the (**a**) inactive and (**b**) activated μ-opioid receptor (MOR) simulations derived using the transition-based reweighting method (TRAM). In each figure, the CVs, [*d*, *α*_*1*_, *α*_2_], have been projected onto the *d* and *α* dimensions, taking into account the interchangeability of the two protomers by duplicating the trajectories through swapping of the two angular CVs prior to the free energy calculation. Dark red/light orange color coding is matched to lowest/highest free-energy values. The insets in the center refer to surface representations of the inactive and activated MOR structures as seen from the extracellular side, with the transmembrane helix 1 (TM1) depicted in blue TM2 in red, TM3 in dark gray, TM4 in orange, TM5 in yellow, TM6 in green, and TM7 in magenta.

Figure 1 shows the free energies of the relative positions of the MOR protomer pairs obtained from the TRAM analysis. Two highly populated regions, clustered around α_1_, α_2_ ≈ 0° and α_1_, α_2_ ≈ π and below distances of 4.5 nm for either inactive or activated MOR conformations suggest a preference for MOR homodimerization through TM4,5,6,7 or TM1,2,H8 contacts, respectively. Notably, the region around α_1_, α_2_ ≈ π presents a broader distribution in the inactive MOR simulations (see larger dark red area in Fig. 1a) compared to those of the activated MOR (Fig. 1b), with the region near TM4 (orange helix in the insets of Fig. 1) of the activated MOR structure less populated compared to that of the inactive MOR.

In order to further elucidate the preferred protomer orientations within putative dimeric configurations of MOR, the microstates of the MSM produced by the TRAM analysis were clustered using the PCCA+ algorithm. Specifically, this method allows for the separation of long- and short-lived microstates and distinguishes the unbound (i.e., monomeric) microstates from the longer-lived dimeric or dimer-like microstates (for details, see Methods section).

**Figure 2:**
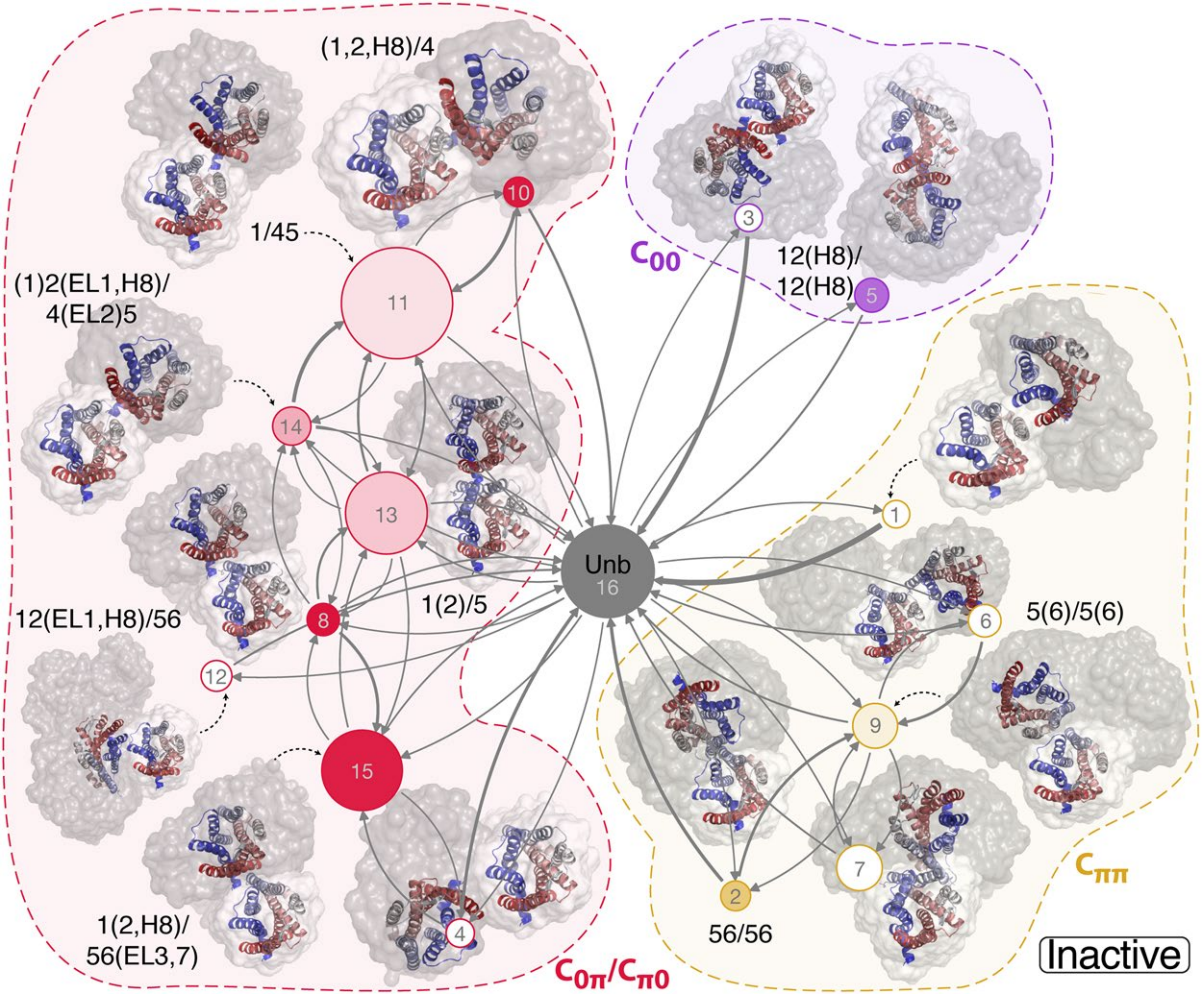
Kinetic networks of inactive MOR macrostates within asymmetric and symmetric components. Asymmetric components *C*_*0π*_ (α_1_ ≈ 0, α_2_ ≈ π) and *C*_*π0*_ (α_1_ ≈ π, α_2_ ≈ 0) are grouped together and shown in red while symmetric components *C*_*ππ*_ (α_1_ ≈ π, α_2_ ≈ π) and *C*_*00*_ (α_1_ ≈ 0, α_2_ ≈ 0) are shown in yellow and purple, respectively. Each node represents a macrostate consisting of a group of PCCA clusters that are spatially and kinetically linked, and whose area is proportional to the probability of the state. The nodes are shaded according to the fraction of dimeric microstates (i.e., those characterized by a dimer population larger than 90%), with a solid color representing a fraction of 100%, and white signifying a fraction of 0%. These macrostates are further labeled according to the interfaces obtained from the contact map analysis of the dimeric microstates. The arrow thickness is proportional to the transition probability between the connected macrostates, ranging between ~3 × 10^−5^ (thinnest line) and 0.04 (thickest line). Representative configurations of the dimeric microstates are shown in cartoon models with the inter-protomeric variability within each macrostate captured by 100 randomly selected frames from that macrostate shown as gray clouds.

**Figure 3:**
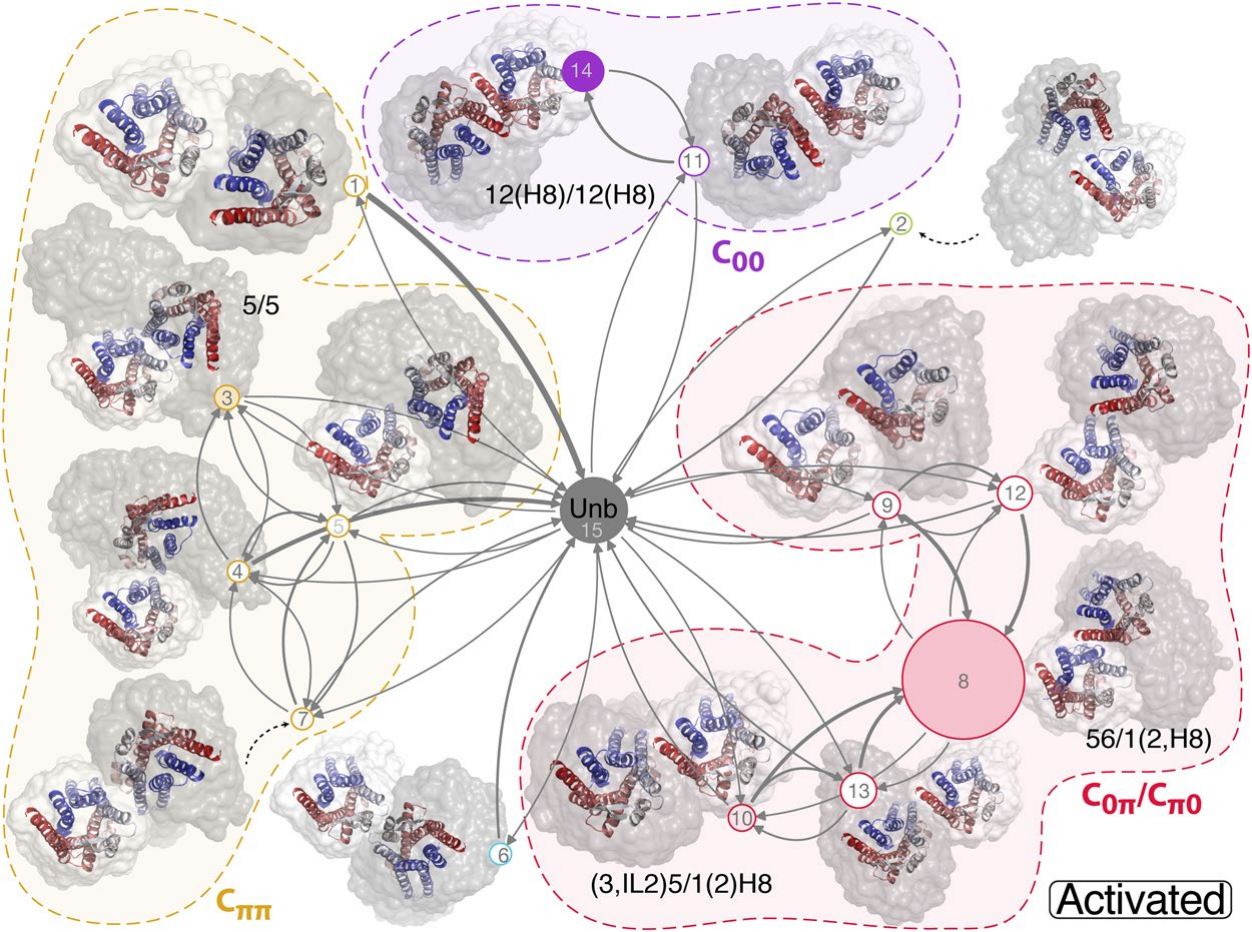
Kinetic networks of active MOR macrostates within asymmetric and symmetric components. Asymmetric components *C*_*0π*_ (α_1_ ≈ 0, α_2_ ≈ π) and *C*_*π0*_ (α_1_ ≈ π, α_2_ ≈ 0) are grouped together and shown in red while symmetric components *C*_*ππ*_ (α_1_ ≈ π, α_2_ ≈ π) and *C*_00_ (α_1_ ≈ 0, α_2_ ≈ 0) are shown in yellow and purple, respectively. Two additional macrostates that do not belong to any component are shown as green and blue circles, respectively. Each node represents a macrostate consisting of a group of PCCA clusters that are spatially and kinetically linked, and whose area is proportional to the probability of the state. The nodes are shaded according to the fraction of dimeric microstates (i.e., those characterized by a dimer population larger than 90%), with a solid color representing a fraction of 100% and white a fraction of 0%. These macrostates are further labeled according to the interfaces obtained from the contact map analysis of the dimeric microstates. The arrow thickness is proportional to the transition probability between the connected macrostates, ranging between 3.7 × 10^-5^ (thinnest line) and ~0.08 (thickest line). Representative configurations of the dimeric microstates are shown in cartoon models with the inter-protomeric variability within each macrostate captured by 100 randomly selected frames from that macrostate shown as gray clouds.

Removing all microstates of the monomeric cluster from the transition matrix revealed four main kinetically disconnected regions for both the inactive and activated MOR systems (see Figs 2 and 3, respectively). These regions, herein referred to as components, encompass spatially and kinetically linked PCCA clusters of microstates between which there are no direct transitions; instead, the progression from one component to another requires passage through the monomeric PCCA cluster. These four main components represent four possible protomer-protomer orientations, two asymmetric – i.e., (α_1_ ≈ 0, α_2_ ≈ π) and (α_1_ ≈ π, α_2_ ≈ 0) – and two symmetric – i.e., (α_1_ ≈ 0, α_2_ ≈ 0) and (α_1_ ≈ π, α_2_ ≈ π) – referred to as C_0π_, C_π0_, C_00_, and C_ππ_, respectively. The two asymmetric components, C_0π_ and C_π0_, whose structures can be grouped together given that protomers are identical, encompass a much larger population than the symmetric components (see red nodes in Figs 2 and 3), forming 87% and 89% of the populations of the bound-like PCCA clusters for the inactive and activated MOR systems, respectively. The symmetric component C_00_ (purple nodes in Figs 2 and 3) forms 2% and 9% of the total bound-like population in the inactive and activated MOR systems, respectively, while C_ππ_ (yellow nodes in Figs 2 and 3) represents 11% and 2% of the population in the same systems. While these four components encompass all microstates of the inactive MOR system with the exception of the unbound states (Fig. 2), two small additional clusters of microstates (0.1% and 0.06% of the stationary occupancy; blue and green circles in Fig. 3) are identified in the activated MOR system. Within each component, the MOR protomers sample a broad range of orientations, which can be further grouped according to the PCCA clusters within those components. Specifically, by imposing symmetry over α_1_ and α_2_, 16 and 15 groups of receptor configurations, referred to as macrostates, were obtained for the inactive and activated MOR systems, respectively (see Methods section for further details).

In order to discriminate between compact dimeric complexes of MOR and loosely bound protomer pairs, we enforced a definition of dimeric states similar to that used in references^16^ and^18^. Specifically, bound MOR protomer pairs were considered to be fully formed dimers whenever each protomer had at least 10 inter-protomer contacts within a distance of 0.8 nm. A dimeric microstate was defined as any microstate for which the probability to observe a dimeric configuration is higher than 90%. Using this definition, we calculated contact maps for all dimeric microstates within each macrostate, and report the different dimeric interactions in each macrostate in Supplementary Figs 2 and 3 for inactive and activated MOR, respectively. Corresponding structures for each interface are depicted in Supplementary Figs 4 and 5. Receptor domains participating in the interface identified at this stage were used to label their corresponding macrostates in the kinetic networks presented in Figs 2 and 3. Specifically, any receptor region with more than three contacts is listed as part of the interface name and regions with less than three contacts are presented in parentheses.

For both active and inactive MOR structures, we observe that each component (the asymmetric components C_0π_ and C_π0_, and the two symmetric components, C_00_ and C_ππ_) is populated with dimeric microstates. While some of the dimeric interfaces are common to the active and the inactive receptors, others are specific to one conformation only, as can be seen in Figs 2 and 3. Within the asymmetric components (red clusters in Figs 2 and 3), both MOR structures form TM5,6/TM1,2,H8 and TM5/TM1,2,H8 interfaces whereas interfaces involving TM4 (e.g., TM4,5/TM1,2,H8) appear to be unique to the inactive MOR. Out of the two symmetric components, the C_ππ_ component (yellow clusters in Figs 2 and 3) yields distinct interfaces for the inactive and activated MOR structures. While the active MOR structure is only able to form dimeric TM5/TM5 interfaces, the two dimeric macrostates that are found in the C_ππ_ component of inactive MOR exhibit TM5,6/TM5,6 interfaces that are similar to those seen in the MOR inactive crystal structure (see Supplementary Figs 4 and 5). Finally, simulations of both the inactive and activated MOR systems yield dimers exhibiting the same TM1,2,H8/TM1,2,H8 interface (C_00_ component, purple clusters in Figs 2 and 3) seen in the inactive crystal structures of MOR and KOR.

To elucidate which residues are the most relevant to the dimerization process, we calculated the average number of contacts formed by each residue over the complete set of microstates. The residues involved in an average number of contacts larger than 0.2 are listed in Supplementary Table 2 and their spatial distribution across the inactive or active MOR structures is shown in Supplementary Fig. 6. In the inactive MOR system, these residues belong to TM1, TM2, TM5, and H8, as well as IL2, IL3, EL1, and EL2, whereas in the active MOR system additional TM6 residues, but not residues in IL2 and EL2, are among those with the highest average numbers of contacts.

For the active MOR system, the three residues with the highest average contact numbers belong to H8, and they are: F350, C351, and I352. While F350 and C351 have significant, albeit slightly lower, average contact numbers in the inactive receptor, I352 has the highest average contact number in this receptor state. In TM1, the residues with the highest average contacts formed are A73^1.37^, S76^1.40^, and I69^1.33^ for the inactive system, and I69^1.33^, V80^1.44^, and M72^1.36^ for the active system (amino acid numbers correspond to the mouse MOR sequence while superscripts refer to the Ballesteros-Weinstein numbering scheme^24^). In TM2, V126^2.62^, L129^2.65^, and M130^2.66^ present the largest average number of contacts formed in both the inactive and active structures. In TM5, the residues most likely to form contacts are I238^5.44^, M243^5.49^, and Y227^5.33^ in the inactive system, or L257^5.63^, R258^5.64^, and Y227^5.33^ in the active receptor. In IL3, S261 and K260 can form more than 0.2 contacts on average in the active system, while in the inactive system only K260 presents an average contact number above this threshold. In the active MOR system, P134 and F135 in EL1 exhibit high contact numbers, while in the inactive receptor, only P134 is above the cut-off of 0.2 contacts. Unique to the TM6 of the active MOR system, residues I298^6.53^, I302^6.57^, and L305^6.60^ are most likely to form inter-protomer contacts with averages above 0.2 contacts. Exclusive to the inactive system, K174 and H171 in IL2, and H223 and P224 in EL2 form above 0.2 contacts on average.

Since the average number of inter-protomer contacts formed by each of the aforementioned residues does not provide information about which two residues are in contact, we also calculated the probability of occurrence for each contact over all microstates. Supplementary Table 3 lists all inter-protomer contacts with probabilities larger than 0.1 calculated for the inactive and active MOR systems. For the active system, the most likely contacts are formed between H8 and TM5 or IL3 with probabilities over 0.25, followed by contacts between TM6 and TM1 or TM2 with probabilities between 0.13 and 0.22. Both groups of contacts fall within the asymmetric component, C_0π_ (red clusters in Fig. 3), which is the most populated component. Specifically, the contacts in dimers of active MOR with the highest probabilities are between IL3 and H8 (i.e., S261(IL3)-I352/F350/C351/E349(H8), and K260(IL3)-I352/C351/F350(H8)), and between TM5 and H8 (i.e., L257^5.63^-C351/F350/I352(H8) and R258^5.64^-F350/C351(H8)). For the inactive system, the most likely contacts also belong to the asymmetric component, C_0π_ (red clusters in Fig. 2), and those with highest probability are: K174(IL2)-I352(H8), M130^2.66^-Y227^5.33^, and A73^1.37^-I238^5.44^.

### Kinetic models of dimerization of inactive or activated MOR receptors

A k-means discretization with ~1500 centers and a lag time of 50 ns was used to build a MSM from TRAM. In order to validate this MSM, we checked for the convergence of the implied timescales at lag times of 20 ns, 50 ns, and 100 ns (see Supplementary Fig. 4). Specifically, timescale distributions at each of these lag times were calculated by bootstrap sampling over the unbiased simulations only. Additionally, the Chapman-Kolmogorov test, which compares the evolution of the probability calculated with the TRAM kinetic model with the one from simulations, was applied at the macrostate level for all transitions from the unbound macrostate to the bound macrostates (Supplementary Fig. 8) to further assess the reliability of the Markovian assumption.

To better differentiate the MOR dimer interfaces (shaded nodes in Figs 2 and 3), we chose to study the kinetics of the system at the macrostate instead of the microstate level with each macrostate representing groups of spatially and kinetically linked PCCA clusters of microstates. A reduced transition matrix was built for this simplified grouping using the validated approach described in^25^ (see Methods section).

The kinetic networks of the macrostates for the inactive and activated MOR systems are shown in Figs 2 and 3, respectively. The macrostates have all been numbered and those containing dimers have been assigned a dimeric interface label that depends on the number of contacts formed. Transition path analysis was then used to obtain the partitioning of the total flux between the monomeric state of MOR and macrostates that contain dimeric interfaces. As shown by this partitioning (see Supplementary Tables 4 and 5 for inactive and active MOR, respectively), while some dimeric interfaces form directly from the unbound state (macrostates #16 and #15 for inactive and active MOR, respectively), others transition through multiple intermediates, suggesting a different dimerization mechanism depending on the interface formed. For instance, in the inactive system, the TM1,2,(H8)/TM1,2,(H8) interface forms directly from the monomeric state with a probability of 94% (see Supplementary Table 4) and only 5% through a loosely bound intermediate (indicated as cluster #3 in Fig. 2). On the other hand, interfaces involving helices TM4, TM5, or TM6, form through metastable intermediates with a higher probability. For instance, while the TM5,6/TM5,6 complex (C_00_) forms directly with a probability of 64% (see Supplementary Table 4), 25% of the flux goes through the TM5,(6)/TM5,(6) state and 7% visits both the TM5,(6)/TM5,(6) state and the loosely bound state #7 in Fig. 2. Notably, states in the asymmetric components have the most heterogeneous dynamics with interfaces between TM1,2 and TM4,5, or TM5,6 freely interchanging in inactive MOR (see Supplementary Table 4).

While certain interfaces form similarly (directly or indirectly) in the inactive and active MOR systems, others exhibit a different dynamic behavior in the two systems. For instance, while the TM1,2,(H8)/TM1,2,(H8) dimer assembles directly in the inactive MOR, it forms through an intermediate state (#11 in Fig. 3) with a probability of 99% (Supplementary Table 5) in the active MOR. On the other hand, interfaces involving helix TM5 (in the symmetric C_ππ_ and the asymmetric components) mostly occur through metastable intermediates in both inactive and active MOR systems (see Supplementary Table 5).

### Concentration dependence of the kinetic rates and fractions of MOR homodimers

Since the concentrations used in the simulations reported in this work are much larger than most experimental concentrations (specifically, 1.37 × 10^4^ µm^–2^ compared to the typical experimental range of 1–100 μm^−2^), we calculated the protein concentration dependence of MOR dimerization kinetics. Briefly (see *Methods* for further details), we describe the kinetics as a two-step reaction:

$$A+B\,\begin{array}{c}{k}_{-1}\\ \leftrightarrows \\ {k}_{+1}\end{array}\,A\cdot B\,\begin{array}{c}{k}_{ji}\\ \leftrightarrows \\ {k}_{ij}\end{array}\,AB$$

where *A* + *B* is the fully dissociated protomeric state, *A•B* is the encounter complex, *AB* is the proper complex, and *k*_*ji*_ and *k*_*ij*_ stand for the rates obtained from the TRAM transition matrix. *k*_−*1*_ and *k*_+*1*_ are second- and first-order diffusive rates which depend on the protomers’ relative 2D diffusion constant D and the 2D concentration *c* as described in^26^. The overall dimerization rate is *k*_*on*_ = *γ k*_+*1*_ where the capture probability *γ* is the probability to form an associated state *AB* from the encounter complex, instead of dissociating to *A* + *B*^27,28^.

In order to compare the kinetics of the main components, the TRAM transition matrix was simplified, further aggregating the macrostates into the *C*_*00*_, *C*_*ππ*_, *C*_*0π*_ and *C*_*π0*_ components. The reduced transition matrix was validated by comparing its implied timescales to the timescales of the full transition matrix of the TRAM model (see Supplementary Fig. 9). The rates *k*_*ji*_ and *k*_*ij*_ were calculated for the four components from the reduced transition matrix.

**Figure 4:**
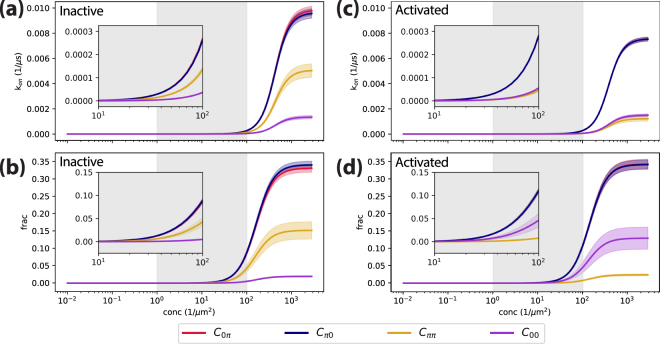
Concentration dependence of the *k*_*on*_ values (panels a and c for the inactive and active MOR, respectively) and dimeric fractions (panels b and d for the inactive and active MOR, respectively). The gray regions indicate the typical concentration ranges commonly used in experiments. The insets refer to concentration values between 10 and 100 μm^−2^.

The concentration dependence of the association rates and fractions of homodimers of activated or inactive MOR structures, obtained using a value of the diffusion constant of 0.1 μm^2^/s (see Discussion), are shown in Fig. 4. As shown in this figure, for concentrations lower than 10^2^ μm^−2^, *k*_*on*_ ~ 0, suggesting that MOR dimerization is negligible at these concentrations. However, at concentrations above 10^3^ μm^−2^, which include the concentrations used for the simulations presented herein, the conversion of the encounter complex *A•B* to the dimeric configurations becomes the rate-limiting step, i.e., the dimerization becomes reaction-limited, and the *k*_*on*_ values converge to different values depending on the dimeric component of the inactive or activated MOR structure. Specifically, the *k*_*on*_ values of the asymmetric components (*C*_*0π*_ and *C*_*π0*_) converge to ~10 ± 0.4 ms^−1^ and ~8.0 ± 0.2 ms^−1^ for the inactive and activated MOR structures, respectively. These values are significantly larger than the *k*_*on*_ values of the symmetric components, *C*_*ππ*_ and *C*_*00*_, which converge to ~5.0 ± 0.5 ms^−1^ and ~1.0 ± 0.2 ms^−1^, respectively, for the inactive MOR system and ~1.0 ± 0.2 ms^−1^ and ~1.0 ± 0.1 ms^−1^, respectively, for the activated MOR structures.

The bound states of both the inactive and activated MOR systems reach a total fraction of ~0.85 ± 0.04 considering the sum of all four components. Notably, the faster rate at which asymmetric complexes contributes to the larger fraction of dimers in the two C_*0π*_ and C_*π0*_ components. In the inactive MOR, these components reach a cumulative fraction of ~0.7 ± 0.02, while the symmetric *C*_*ππ*_ and *C*_*00*_ components are at ~0.15 ± 0.02 and ~0.02 ± 0.002, respectively, as shown in Fig. 4a. Similar to the inactive MOR system, the asymmetric components have a cumulative fraction of ~0.7 ± 0.02 in the activated receptor whereas opposite values (0.02 ± 0.003 for *C*_*ππ*_ and ~0.13 ± 0.03 for *C*_*00*_) are obtained for the symmetric components (see Fig. 4b).

As the concentration decreases to the upper limit of the typical experimental range at 10^2^ μm^−2^, the *k*_*on*_ values reach ~0.3 ms^−1^ for the asymmetric components (*C*_*0π*_ or *C*_*0π*_) of both the inactive and activated MOR structures with dimer fractions of ~9% and ~12%, respectively. The *C*_*ππ*_ component of the inactive MOR dimers reaches a *k*_*on*_ value of 0.1 ms^−1^ with a dimer fraction of ~0.05, while the *C*_*00*_ conformations become negligibly small. For the activated MOR dimers, *k*_*on*_ values for both symmetric components are ~0.05 ms^−1^ at this concentration reaching dimer fractions below 0.05. The *k*_*on*_ calculated for receptor concentrations of 10^2^ μm^−2^ and dimer lifetime values (see Methods section for details) are presented in Table 1 for each main component of the inactive and active MOR systems. The lifetimes of the asymmetric components are ~220 μs and ~280 μs for the inactive and active MOR systems, respectively. Among the symmetric components, *C*_*ππ*_ has a lifetime of 130 μs and 100 μs for the inactive and active MOR systems, respectively, while *C*_*00*_ exhibits dimer lifetimes of 90 μs and 380 μs for the same systems.

**Table 1:** The *k*_*on*_ values (calculated for protein densities of 10^2^ μm^−2^) and dimer lifetimes of the four kinetic components, *C*_*0π*_, *C*_*π0*_, *C*_*ππ*_, and *C*_*00*_, derived for the inactive and active MOR systems.

Kinetic Components	Inactive MOR		Active MOR	
	*k*_*on*_ (μs^−1^ μm^−2^)	*T*_*off*_ (μs)	*k*_*on*_ (μs^−1^ μm^−2^)	*T*_*off*_ (μs)
C_0π_	3 × 10^−6^ ± 6 × 10^−8^	210 ± 20	3 × 10^−6^ ± 5 × 10^−8^	280 ± 20
C_π0_	3 × 10^−6^ ± 6 × 10^−8^	220 ± 20	3 × 10^−6^ ± 5 × 10^−8^	280 ± 20
C_ππ_	1 × 10^−6^ ± 1 × 10^−7^	130 ± 30	5 × 10^−7^ ± 8 × 10^−8^	100 ± 20
C_00_	4 × 10^−7^ ± 3 × 10^−8^	90 ± 10	6 × 10^−7^ ± 4 × 10^−8^	380 ± 60

### MOR dimerization assessed by FRET acceptor photobleaching experiments

**Figure 5:**
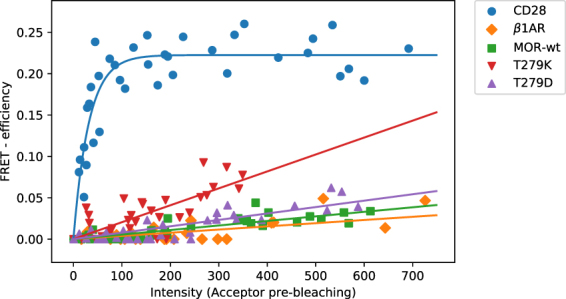
FRET-efficiencies of SNAP-labeled MOR constructs plotted as a function of acceptor intensity (i.e., expression level) before photobleaching. CD28 (blue color) is used as reference dimer whereas β1AR (orange color) is used as reference monomer. Wild-type MOR (green color), as well as inactive MOR mutant T279D (purple color) and constitutively active MOR mutant T279K (red color), all show an unspecific increase in FRET efficiencies.

To experimentally assess the degree of MOR dimerization in a concentration-dependent manner, we conducted acceptor photobleaching experiments to determine FRET efficiencies between mouse MOR pairs (see Supplementary Fig. 10). Specifically, SNAP-tagged MOR constructs were selectively labeled with an equimolar combination of SNAP-Surface 549 (SS549) and SNAP-Surface Alexa Fluor 647 (SS647) on the outer cell membrane. This FRET pair has a sufficient spectral overlap, which leads to an estimated Förster-distance^29^ of 74 Å. The donor (SS549) is independently photostable and is not significantly affected during acceptor photobleaching (Supplementary Fig. 11). A construct coding for a dimeric cell-surface receptor^30^, CD28, was used as a reference dimer. As expected and shown in Fig. 5, this construct shows expression dependence, fast increase, and saturation of FRET efficiencies, with a k_d_ = 22.08 ± 6.2 relative fluorescent units (RFU), and a FRET efficiency max = 22.25 ± 0.6%. Based on previously published single-molecule experiments^15^ suggesting a low degree of di-/oligomerization (monomers or dimers) for the β_1_-adrenergic receptor (β1AR), we used this system as a reference monomer in the experiments reported here. As expected, β1AR showed a quite low linear (unspecific) increase of FRET efficiencies (Fig. 5) with the highest obtained FRET efficiency of 4.7% at an expression level of 726 RFUs (slope = 0.004%/RFU). Experiments with MOR do not deviate significantly from the β1AR example of a monomer (Fig. 5). The system does not saturate and its highest observed FRET efficiency is 3.4% at 613 RFUs (slope = 0.005%/RFU). To further assess the dependence of the active or inactive state of MOR on its di-/oligomerization, we performed experiments with two MOR mutants: T279K and T279D (Fig. 5). In the inactive MOR crystal structure, the T279^6.34^ residue forms a hydrogen bond with R165^3.50^, which replaces the classical R^3.50^-D/E^6.30^ salt bridge that stabilizes the inactive state of rhodopsin-like GPCRs. Previous studies^31^ show that the T279K mutant is a constitutively active receptor, presumably due to an electrostatic repulsion with R165^3.50^, whereas the T279D mutant is an inactive receptor possibly due to its ability to form a salt-bridge with R165^3.50^. For both mutants, the FRET efficiency also increased linearly (unspecifically) with expression levels as shown in Fig. 5. The inactive T279D mutant exhibited a slightly increased slope (0.007%/RFU) in comparison to the wild-type MOR whereas the constitutively active T279K mutant showed a more significant, but still unspecific, increase in FRET efficiencies with a slope of 0.02%/RFU. For a comparison of the significance values see Supplementary Table 6.

## Discussion

While it is well established that the MOR is functional in its monomeric form^32^, a conclusion has not been reached as to whether it is mostly present as monomers, dimers, or higher order oligomers under physiological conditions *in vivo*. In this work, we report, for the first time, a multi-ensemble Markov model describing the kinetics underlying MOR association and dissociation, and providing predictions of MOR homodimerization rates and fractions at physiological concentrations.

A few cautionary points should be kept in mind while considering the results of this study. Firstly, unlike what happens under physiological conditions, the coarse-grained MARTINI force field used here to simulate MOR prevents any major conformational rearrangement during receptor association. Notwithstanding this limitation, the computational approach presented here provides inferences of MOR dimerization that are verified experimentally through measurements of FRET efficiency. Secondly, since an unfeasible amount of simulation time would be required to observe relevant events such as MOR dimerization at physiological concentrations, the simulations presented here were performed at much higher receptor densities (i.e., ~1.37 × 10^4^ µm^–2^) than those afforded under physiological conditions^33,34^ or commonly used in fluorescence resonance energy transfer (FRET) or spatial brightness experiments (e.g., 0.1 µm^−2^–100 µm^−2^; see for instance^35–37^). Furthermore, the natural cellular environment of MOR is far more crowded and complex than its counterpart in our simulations, which is composed of POPC and CHOL molecules only. Thus, it is not surprising that the diffusion coefficients obtained from the simulations presented here are several orders of magnitude larger (i.e., ~ 10 μm^2^/s) than those observed in experiments (i.e., 0.02 μm^2^/s to 0.2 μm^2^/s)^13,15^, where the MOR movement within the membrane is impaired. It is also important to point out that the diffusion coefficients obtained from the simulations reported here are calculated using portions of the trajectories for which the distance between protomers range between 8 nm to 11 nm. While these values are appropriate given the simulation box size, the diffusive behavior of the protomers is expected to be different at lower concentrations where the distance between them is much larger.

The above considerations prompted us to use monomer diffusion coefficients obtained from experiments rather than from simulations in the diffusive step of the reaction-diffusion model employed to study the concentration-dependence of kinetic rates and fractions of MOR homodimers at physiological concentrations. Using a physiologically relevant diffusion coefficient of 0.1 μm^2^/s, we obtain negligible dimeric fractions of MOR at most experimental concentrations, including physiological concentrations. These homodimers are characterized by relatively short lifetimes that depend on the interface and do not exceed ~0.3 ms. Notably, at the lower end of a typical experimental concentration range (i.e., 0.1–10/μm^2^), MOR dimer populations become negligible even for much larger diffusion coefficients such as those obtained from simulations. This was confirmed by linear increases in FRET efficiencies by acceptor photobleaching, which argued in favor of a very low degree of homomerization for MOR.

Compared to other GPCRs whose dimerization has been studied experimentally, our results suggest smaller populations of MOR homodimers with much shorter lifetimes. For instance, unlike the β_1_-adrenergic receptor, which had been reported to exhibit a low degree of di-/oligomerization by single-molecule experiments^15^, the β_2_AR had been shown to form 60% of dimer and higher order oligomer populations, at concentrations of ~0.25/μm^2^ and with lifetimes on the order of a few seconds^15^. Similarly, the M_1_ muscarinic receptor had been shown to form ~30% dimers at concentrations of ~1.8/μm^2^ with lifetimes on the second time-scale^13^. Our computations suggest that MOR dimer populations are much lower at these concentrations, suggesting a weaker propensity for MOR dimerization compared to β_2_AR and M_1_ muscarinic receptor. Additionally, in the case of the β_2_AR, agonist binding showed no significant effect on dimerization^15^ while our results suggest a small increase in dimer fractions upon activation at concentrations near 10^2^/μm^2^. However, this difference becomes irrelevant at smaller and more physiological concentrations, as confirmed by FRET acceptor photobleaching measurements of the inactive T279D and constitutively active T279K mutants of MOR.

Notably, the differences observed in the dimerization kinetics of the inactive and activated MOR systems are accompanied by distinct dimer interfaces. The available inactive and active MOR crystal structures suggest that while dimerization can occur at the symmetric interface of TM1,2,H8/TM1,2,H8 for both systems, the outward movement of the TM6 in the MOR activated crystal structure would prevent the TM5,6/TM5,6 interface from occurring^38^. The interfaces formed in the simulations presented here corroborate these conjectures and furthermore suggest that for the active MOR structure a TM5/TM5 interface might also be possible. Unlike our earlier simulation results in which we found the TM4/TM4 interface to form with high probability between active MOR protomers, we only observed here the TM5/TM5 interface^16^. In contrast, the TM1,2,H8/TM1,2,H8 interface, which has been also inferred by the inactive MOR and KOR crystal structures^38,39^, also formed in our previous simulations, albeit with much lower probability.

Compared to the symmetric interfaces, our results suggest that asymmetric interfaces of MOR dimers have significantly higher propensities to form, in line with our earlier results^16^. While asymmetric dimers of the inactive MOR involving the TM1,2,H8/TM4, TM1,2,H8/TM4,5 and TM1,2,H8/TM5 interfaces were heavily favored in both earlier and present simulations, the TM1,2,H8/TM5,6 interface was observed only in the simulations reported here. Furthermore, the small population of dimer interfaces involving TM7 observed in the earlier study are not observed in the current simulations, nor is the TM4/TM5 interface. On the other hand, the active MOR structures also have high propensities for the asymmetric dimer interfaces of TM1,2,H8/TM5,6 and TM1,2,H8/TM5. The TM1,H8/TM6,7 interface observed in CXCR4^40^ and the TM4/TM7 interface of CCR5^41^ are absent in the simulations of both inactive and active MOR despite having been reported to form ~8% of the dimer population in our earlier results.

Predictions of residues involved in dimeric contacts suggest several potential mutations that can significantly affect the dimerization process. For both the inactive and active systems, we found that H8 includes some of the residues with the largest average contact numbers as well as being involved in some of the most likely contacts. For instance, I352 in H8 forms an average of 1.48 and 1.3 contacts in the active and inactive systems, respectively, while participating in the top 3 most likely contacts that appear in both systems, between IL3 and H8 in the active MOR system and between IL2 and H8 in inactive MOR. Similar to I352, F350 and C351 in H8 are also involved in putative dimeric interfaces of MOR, in addition to playing a significant role in TM5 and H8 contacts in the active MOR system. This analysis also draws attention to M130^2.66^ as a potential point of mutation, since it plays an important role in TM5-TM2 and TM2-EL2 contacts in the inactive MOR system, in addition to TM6-TM2 contacts in the active MOR. Notably, L129^2.65^ is likely to have an effect similar to M130^2.66^. Among the TM1 residues involved in dimerization, A73^1.37^ plays an important role in TM5-TM1 contacts in the inactive MOR system, while I69^1.33^ participates in prominent TM1-TM6 contacts. Combined, the appropriate mutations at these sites might enhance the dimerization propensities of the MOR system by modulating contacts that are crucial to the formation of asymmetric interfaces, which are suggested to be the most likely candidates for dimerization according to our results.

In conclusion, our study presents a computational framework that elucidates, for the first time, the dimerization kinetics of MOR by combining unbiased and biased coarse-grained simulations coupled with multi-ensemble Markov modeling and a reaction-diffusion model, which provides details both at the molecular and the ensemble scale. Our computational results suggest that the MOR does not dimerize at concentrations used in most experimental methods in contrast to other prototypic GPCRs, such as the β_2_-adrenergic receptors and the M_1_ muscarinic receptor. These results are supported by FRET acceptor photobleaching experiments, which confirm that MOR does not exhibit concentration-dependent specific dimerization over a wide range of expression levels. In striking contrast to CD28, MOR exhibits a linear (unspecific) increase in FRET efficiencies rather similar to the largely monomeric β1AR^15^. Moreover, FRET measurements performed on both the inactive T279D mutant receptor and the constitutively active T279K mutant suggested that MOR dimeric fractions might be independent on a specific conformational state of the receptor. Notably, the slope of FRET efficiencies was significantly higher for the constitutively active T279K mutant compared to the inactive T279D mutant and wild-type MOR. This might be due to either an enhanced propensity of this mutant receptor to form true complexes from encounter complexes or to its enhanced internalization, which leads to non-specific aggregation through clustering in clathrin coated pits and vesicles. In summary, our study provides a quantitative explanation for some conflicting experimental results on GPCR oligomerization.

## Methods

### System set-up

All non-receptor atoms of the crystal structures of inactive (PDB ID: 4DKL^38^) and activated (PDB: 5C1M^42^) MOR were removed, including co-crystallized ligands, the T4L fusion protein in the inactive structure, and the G protein mimetic nanobody in the activated structure. The missing intracellular loop 3 (ICL3) in the inactive MOR structure was built by homology modeling using MODELLER^43^ and the high-resolution crystal structure of DOR^44^ as a template. The N-terminal segment of the activated structure was removed and the remaining residues of H8 added so that each structure consisted of sequence residues 65 to 352. The *martinize* python script was used to coarse grain the structures within the MARTINI v2.2 force field^19–22^. A modified version of the elastic network was applied to maintain the receptor tertiary structure^45,46^. Specifically, a harmonic force was applied to all backbone (BB) bead pairs with relative distance less than 0.9 nm. A force constant of 1000 kJ mol^−1^ nm^−2^ was used when both beads belonged to regions with helical secondary structure while 250 kJ mol^−1^ nm^−2^ was used when either bead was in a loop region. Two inactive or activated MOR protomers were randomly rotated around the z-axis and placed in a 15 × 15 × 11 nm^3^ box along the diagonal such that the COM of the receptors were approximately 6.25 nm apart. The receptors were then embedded in a POPC/10% CHOL membrane constructed using the *insane* python script^47^. Each system contained approximately 576 POPC and 64 CHOL molecules. A total of 704 dimer/membrane complexes were generated: 352 for the inactive MOR and 352 for the activated receptor. Each complex was solvated and water and ions were added to neutralize the charge.

### Simulations in the unbiased ensemble

Up to 10,000 steps of energy minimization were performed on each system followed by 500 ns of equilibration keeping position restraints on the BB beads of the receptor. The position restraints were slowly removed by performing four 50 ns runs with gradually decreasing force constants (k = 500, 100, 50, 10 kJ/mol/nm^2^). Each of the 704 receptor/membrane complexes was then run for at least 5 µs. All simulations were performed using Gromacs 5.0^48^ and in the NPT ensemble at 310 K and 1 bar. Constant temperature and pressure were maintained using the velocity-rescale^49^ and Berendsen^50^ algorithms, respectively. The Coulombic interactions decayed smoothly to zero between 0 and 1.2 nm while the van der Waals interactions decayed to zero between 0.9 and 1.2 nm.

### Collective variables and preliminary kinetic model

The results were first analyzed by describing the system with a three-dimensional order parameter comprising (i) the distance *d* between the protomers’ COM, (ii) the angle α_1_ formed by the COM of one protomer, the COM of the second protomer, and the COM of its transmembrane (TM) helix 1, and (iii) the corresponding angle α_2_ obtained swapping the two protomers. To efficiently account for angle periodicity, the four-dimensional order-parameter (d*sin*α_1_, d*cos*α_1_, d*sin*α_2_, d*cos*α_2_) was used in the calculations. An initial kinetic model was set up using this order parameter and discretizing the trajectories into 6000 microstates using k-means clustering for all d < ½ L, and using a lag-time of 50 ns. Configurations with the protomers at a distance d ≥ ½ L were assigned to a separate microstate. To improve statistics and account for the interchangeability of the protomers in a homomeric system, each unbiased trajectory was duplicated with swapped values of the angles α_1_ and α_2_, corresponding to exchanging the two proteins. The transition matrix was estimated by maximum likelihood from the count matrix for both the active and inactive systems, and subsequently coarse-grained using PCCA+ to obtain 37 clusters for both the inactive or active systems, respectively. The number of clusters was chosen as the smallest value with at least one macrostate including only microstates with *d* > 4.2 nm and with a flat angular distribution, which corresponded to the unbound state. One unique cluster with these properties was identified for both active and inactive systems. Removing this macrostate from this preliminary transition probability matrix resulted in five main disconnected sets of clusters in the case of the inactive MOR system and six sets for the activated MOR. Once the system enters in one of such disconnected clusters, it cannot transition into another one without first passing through the unbound cluster. K-means clustering, transition matrix estimation and spectral clustering were performed using pyemma (version 2.3)^51^ python functions.

### Simulation details for umbrella sampling runs

To aid convergence of the free-energy and kinetic estimation, umbrella sampling runs were performed restraining the system around selected k-mean centers. Specifically, to select the centers for biased sampling, Transition Path Theory (TPT) from the microstate with d ≥ ½L to the most probable microstate in each of the disconnected components including bound structures, was applied to the preliminary kinetic model described above, and the k-mean centers of microstates with a committor probability p > 10% were selected as umbrella positions. Overall, 314 and 323 umbrella centers were selected for the active and inactive receptor systems, respectively. The position of the centers of umbrella sampling runs is displayed in Supplementary Fig. 12. Umbrella sampling simulations were run for 0.3 μs, using the same parameters of the unbiased simulations, and applying harmonic restraints with elastic constants of k_d_ = 250 kJ/mol/nm^2^ and k_α_ = 100 kJ/mol, respectively, for the distance and angle components of the order parameter. For a small set of runs the elastic constants were reduced to k_d_ = 100 kJ/mol/nm^2^ and k_α_ = 80 kJ/mol, respectively. Restraints were applied using the Plumed 2.1 plugin^52^.

### Markov state modeling and multi-ensemble Markov models

The data from the unbiased and biased simulations were combined using the transition-based reweighting analysis method (TRAM)^23^. To reduce the number of microstates in the calculation, microstates in the unbound PCCA+ component of the preliminary kinetic clustering that did not show transitions out of the component in the 50 ns-lag count matrix were resampled, reducing their number from 5214 to 703 and from 5412 to 911 for the inactive and active MOR systems, respectively. The resulting k-means clustering comprised 1490 microstates in total for both the inactive and active receptor systems. The final multi-ensemble model was estimated using the TRAM algorithm and a convergence threshold based on the maximal free energy change of 10^−9^. Further decreasing the convergence threshold to 10^−12^ resulted in transition matrices without appreciable differences from those with relaxed convergence criteria. The implied timescales from the transition matrix for the unbiased thermodynamic state were calculated for multiple lag-times and their convergence checked. Errors on timescales and rates were obtained by bootstrap. Specifically, 10 bootstrap samples were extracted from the data by sampling of the unbiased trajectories and the TRAM estimator was used to obtain a distribution of transfer matrices. Best estimate and confidence intervals are reported using the mean and the 5% and 95% percentiles for the microstate free-energies and timescales.

### Coarse-graining of the transfer matrix

To obtain kinetic information at a more coarse-grained level, the final TRAM transition matrix was clustered using the PCCA+ algorithm and 51 or 35 clusters for the inactive and activated MOR structures, respectively. The number of clusters to be used was decided using the same criteria applied in the case of the unbiased MSM. Furthermore, the approach used to find disconnected components in the unbiased MSM was repeated for the TRAM transition matrices, resulting in four main disconnected components for both systems and two additional components of negligible populations for the active system. The four main components correspond to the four possible combinations of the two heavily populated regions of the free energy landscapes centered around values of the angles α_1_, α_2_ ≈ 0 and α_1_, α_2_ ≈ π, respectively. We refer to these groups as the *C*_*00*_ component for the TM1,2,H8/TM1,2,H8 region (centered around α_1_, α_2_ ≈ 0), the *C*_*ππ*_ for the TM4, 5, 6/TM4, 5, 6 region (centered around α_1_, α_2_ ≈ π), and the *C*_*0π*_ component for the TM1, 2,H8/TM4, 5, 6 region (centered around α_1_ ≈ 0, α_2_ ≈ π and α_1_ ≈ π, α_2_ ≈ 0).

The coarse-grained rates connecting the PCCA components were calculated using the approach described in ref.^25^. Briefly, reduced dynamic models defined on the PCCA states were obtained by imposing the same cross-relaxation times observed in the full microscopic model. The coarse-grained transfer matrix ${T}_{IJ}^{CG}$ was obtained by solving

1 $$\sum _{i\in I,\,j\in J}{A}_{ij}^{-1}{\pi }_{j}^{micro}={B}_{IJ}^{-1}{\pi }_{J}^{CG}$$

with

2 $$\{\begin{array}{rcl}{A}_{ij} & = & {\delta }_{ij}+{\pi }_{i}^{micro}-{T}_{ij}^{micro}\\ {B}_{IJ} & = & {\delta }_{IJ}+{\pi }_{I}^{CG}-{T}_{IJ}^{CG}\end{array}$$

where *π*^*micro*^ and *T*^*micro*^ are the microscopic stationary probability and transfer matrix from the TRAM estimation, respectively, and ${\pi }_{I}^{CG}={\sum }_{i\in I}{\pi }_{i}^{micro}$are the coarse-grained probabilities on the PCCA+ components. This procedure results in a coarse-grained Markov model that best emulates the dynamics of the full model at both short and long times. To validate the resulting approximation, the Chapman-Kolmogorov test was performed comparing the time-behavior of the occupation probability of each of the PCCA components from the unbiased simulations to the same quantity calculated using the full transition matrix and the coarse-grained transition matrix.

### Definition of dimeric states and contact map calculation

For each microstate *k*, the probability ${p}_{k}^{(Tot)}(n)$ of observing at least *n* inter-protomer contacts (defined as backbone beads below 0.8 nm) and the probability ${p}_{k}^{(Micro)}(i,\,j)$ of each individual contact being formed were calculated as averages over the frames of the unbiased trajectories. Microstates with a high probability of having at least 10 inter-protomer contacts, i.e. ${p}_{k}^{(Tot)}(n=10)\ge 0.9$, were defined as dimeric microstates. Contact maps for each macrostate K were obtained by averaging ${p}_{K}^{(PCCA,dimeric)}(i,\,j)={\sum }_{k\in K}^{\text{'}}{\pi }_{k}{p}_{k}^{(Micro)}(i,\,j)$ where *π*_*k*_ is the stationary probability for microstate *k* from the converged TRAM model and the prime on the sum indicates it is over all dimeric microstates assigned to the macrostate.

### Reaction-diffusion model

In order to extract from the simulations inferences for different receptor concentrations, we employ the simple kinetic model

3 $$A+B\,\begin{array}{c}{k}_{-1}\\ \leftrightarrows \\ {k}_{+1}\end{array}\,A\cdot B\,\begin{array}{c}{k}_{ji}\\ \leftrightarrows \\ {k}_{ij}\end{array}\,AB$$

where $A\cdot B$ represents an encounter complex and *AB* a proper complex, *k*_+1_ and *k*_−1_ are a second- and first- order diffusive rates that depend on the proteins’ relative 2D diffusion constant D and 2D concentration c, and *k*_*ij*_ are the rates obtained from the coarse-graining of the TRAM transition matrix. The effective on-rate for the dimerization reaction is given by *k*_*on*_ = *γk*_*+*1_ (e.g., see^27^) where *γ* is the capture probability $\gamma ={\sum }_{j}{k}_{ij}/({k}_{-1}+{\sum }_{j}{k}_{ij})$, i.e. the probability to form an associated state AB rather than dissociating to A + B, starting from the encounter complex $A\cdot B$. To model the diffusive second-order rate *k*_+1_, we use the approach in ref.^26^ for finite protein concentration, and obtain

4 $${k}_{+1}=\frac{4\pi D}{\mathrm{log}\,\frac{\lambda }{{r}_{0}}}$$

where *λ* = (*πc*)^−1/2^ is the mean-free path at concentration *c* and *r*_0_ is the protein distance in the encounter complex. Here, we model the encounter complex with the dissociated state from the PCCA+ analysis obtained from the simulations, and use the mean distance between protomers in this state ${\sum }_{i\in U}{d}_{i}{\pi }_{i}$, where *π*_*i*_ are the TRAM steady-state probabilities. For the inactive and active system, we obtain *r*_0_ = 6.2 nm and 6.3 nm, respectively. The relative diffusion constant D of the proteins was estimated by fitting the time-dependence of the variance of protein-protein distance (d) distribution at increasing lag times (up to 10 ns) for trajectories starting at d > 80 nm. We obtained similar values D = 10^−8^ μm^2^/ns for the active and inactive receptors, in line with estimates from previous publications with the same force field^53^. Note that this value is not rescaled with the common factor 4 for the Martini forcefield. We calculate the capture probability following the Northrup-Allison-McCammon protocol^28^ as:

5 $$\gamma =\frac{{f}_{{\rm{ass}}}({r}_{1})}{1-{f}_{{\rm{esc}}}({r}_{1}){p}_{{\rm{ass}}}({r}_{1})}$$

where *f*_ass_ is the fraction of trajectories that start with the monomers at distance *r*_0_ and enter an associated state (i.e. any PCCA cluster except the dissociated one) before reaching an “escape” distance *r*_1_, and ${p}_{{\rm{ass}}}=1-(\mathrm{log}\,\lambda /{r}_{1})/(\mathrm{log}\,\lambda /{r}_{0})$ is a factor accounting for the fact that only a fraction of the trajectories that reaches *r*_1_ will eventually escape rather than return to *r*_0_ Values for *f*_ass,esc_ were obtained from the unbiased simulations for *r*_1_ = 7 nm.

To model the concentration dependence of *k*_*on*_ we used the approach in ref.^23^ to calculate the rates from the combined dissociated states (A + B and $A\cdot B$) to the associated states *AB* in the kinetic model; following this approach, *k*_−1_ is obtained from the definition of *γ*. The *k*_*off*_ values are reported as the rates from the associated states to the encounter complex $A\cdot B$. Final times and rates were scaled using a factor 4 to account for faster dynamics in the Martini CG force-field.

### Cell-culture

All experiments were performed with transiently transfected HEK 293 cells (Sigma, Munich, Germany). Cells were cultured in DMEM (Dulbecco’s modified Eagle’s medium) (PAN Biotech, Aidenbach, Germany), supplemented with 4,5 g/L glucose, 2 mM L-glutamin 10% FCS (Biochrome), 100 units/mL penicillin and 0.1 mg/mL streptomycin, at 37 °C, 5% CO2. To split cells, growth medium was removed by aspiration and cells were washed once with 10 ml of phosphate-buffered saline (Sigma), followed by trypsinization for 1–2 minutes in 3-ml of Trypsin 0.05%/EDTA 0.02% (PAN biotech) solution. Cells were routinely tested for mycoplasma infection using PCR mycoplasma test kit (Applichem GmbH, Darmstadt, Germany).

### Microscope sample preparation and SNAP-labeling

Cells seeded on 25 mm coverslips were transfected with SNAP-tagged receptor-constructs using Effectene Transfection Reagent (QIAGEN, Hilden, Germany) according to manufacturer’s protocol. 24–36 hours after transfection cells were labeled with SNAP-Surface Dyes 549 and Alexa Fluor 647 (New England Biolabs, Frankfurt am Main, Germany) according to the manufacturer’s instructions. Briefly, 24 hours after transfection, the culture medium was exchanged with labeling medium (5 µM SNAP-dye in DMEM) followed by a 30 minute incubation at 37 °C. After incubation cells were washed three times with DMEM and immediately taken for imaging in HBSS-Buffer.

### FRET-Acceptor photoBleaching (AB) in confocal microscopy

Prepared samples were placed into an Attofluor™ Cell Chamber (Fisher Scientific GmbH, Schwerte, Germany). Coverslips were washed once with HBSS (Hank’s Balanced Salt Solution) and the chamber was filled with 500 mL HBSS. The chamber was mounted onto a Leica SP8 confocal laser-scanning microscope. Cells were imaged using the Leica FRET-AB wizard with a HC PL APO CS2 40x/1.3 numerical aperture oil immersion objective. A 1.5 mW white light laser was set to 85% and a 560 nm laser line was used at 5% power for the donor imaging. For the acceptor imaging a 652 nm laser line at 2% power was used and for the bleaching step increased to 50% over 10 frames. 512 × 512 pixel images of the bottom cell-membrane expressing SNAP-tagged receptor-constructs were acquired with a hybrid detector in standard mode. Emission of donor channel was recorded within 575–640 nm and acceptor channel was acquired between 658 nm–776 nm. The zoom factor was set to 5.5 x resulting in a pixel-size of 0.103 µm and the laser scanning speed was set to 400 Hz. There were at maximum 2 cells taken for analysis per image. FRET efficiencies were calculated with the manufacturer’s Wizard tool based on the provided formula (see Supplementary Fig. 10). Potential vesicles close to the cell surface were excluded by the drawing of the region of interest.

### Data availability

The data that support the findings of this study are available from the corresponding author upon request.

## Electronic supplementary material

Supplementary Information

## References

[1] Sartania N, Appelbe S, Pediani JD, Milligan G (2007). Agonist occupancy of a single monomeric element is sufficient to cause internalization of the dimeric beta2-adrenoceptor. Cell Signal.

[2] Parenty G, Appelbe S, Milligan G (2008). CXCR2 chemokine receptor antagonism enhances DOP opioid receptor function via allosteric regulation of the CXCR2-DOP receptor heterodimer. Biochem J.

[3] Vilardaga JP (2008). Conformational cross-talk between alpha2A-adrenergic and mu-opioid receptors controls cell signaling. Nat Chem Biol.

[4] Pin JP, Galvez T, Prezeau L (2003). Evolution, structure, and activation mechanism of family 3/C G-protein-coupled receptors. Pharmacol Ther.

[5] Brock C (2007). Activation of a dimeric metabotropic glutamate receptor by intersubunit rearrangement. J Biol Chem.

[6] Salahpour, A. & Masri, B. Experimental challenge to a ‘rigorous’ BRET analysis of GPCR oligomerization. *Nat Methods* **4**, 599–600, author reply 601 (2007). 10.1038/nmeth0807-599 17664941

[7] Lohse MJ (2006). G protein-coupled receptors: too many dimers?. Nat Methods.

[8] Chabre M, le Maire M (2005). Monomeric G-protein-coupled receptor as a functional unit. Biochemistry.

[9] James JR, Oliveira MI, Carmo AM, Iaboni A, Davis SJ (2006). A rigorous experimental framework for detecting protein oligomerization using bioluminescence resonance energy transfer. Nat Methods.

[10] Bouvier, M., Heveker, N., Jockers, R., Marullo, S. & Milligan, G. BRET analysis of GPCR oligomerization: newer does not mean better. *Nat Method*s 4, 3–4, author reply 4 (2007). 10.1038/nmeth0107-3 PMC2246005 17195017

[11] Milligan G (2008). A day in the life of a G protein-coupled receptor: the contribution to function of G protein-coupled receptor dimerization. Br J Pharmacol.

[12] Milligan G (2009). G protein-coupled receptor hetero-dimerization: contribution to pharmacology and function. Br J Pharmacol.

[13] Hern JA (2010). Formation and dissociation of M-1 muscarinic receptor dimers seen by total internal reflection fluorescence imaging of single molecules. Proceedings of the National Academy of Sciences of the United States of America.

[14] Kasai RS (2011). Full characterization of GPCR monomer-dimer dynamic equilibrium by single molecule imaging. J Cell Biol.

[15] Calebiro D (2013). Single-molecule analysis of fluorescently labeled G-protein-coupled receptors reveals complexes with distinct dynamics and organization. Proc Natl Acad Sci USA.

[16] Marino, K., Prada-Gracia, D., Provasi, D. & Filizola, M. Impact of Lipid Composition and Receptor Conformation on the Spatio-Temporal Organization of mu-Opioid Receptors in a Multi-component Plasma Membrane Model. *PLOS Computational Biology* **12** (2016). 10.1371/journal.pcbi.1005240 PMC5154498 27959924

[17] Periole X, Knepp AM, Sakmar TP, Marrink SJ, Huber T (2012). Structural determinants of the supramolecular organization of G protein-coupled receptors in bilayers. J Am Chem Soc.

[18] Provasi, D., Boz, M. B., Johnston, J. M. & Filizola, M. Preferred Supramolecular Organization and Dimer Interfaces of Opioid Receptors from Simulated Self-Association *PLOS Comp. Biol*., accepted (2015). 10.1371/journal.pcbi.1004148 PMC4379167 25822938

[19] de Jong DH (2013). Improved Parameters for the Martini Coarse-Grained Protein Force Field. Journal of Chemical Theory and Computation.

[20] Marrink SJ, de Vries AH, Mark AE (2004). Coarse grained model for semiquantitative lipid simulations. Journal of Physical Chemistry B.

[21] Marrink SJ, Risselada HJ, Yefimov S, Tieleman DP, de Vries AH (2007). The MARTINI force field: Coarse grained model for biomolecular simulations. Journal of Physical Chemistry B.

[22] Monticelli L (2008). The MARTINI coarse-grained force field: Extension to proteins. Journal of Chemical Theory and Computation.

[23] Wu H, Paul F, Wehmeyer C, Noé F (2016). Multiensemble Markov models of molecular thermodynamics and kinetics. Proceedings of the National Academy of Sciences of the United States of America.

[24] Ballesteros JA, Weinstein H (1995). Integrated methods for the construction of three-dimensional models and computational probing of structure-function relations in G protein-coupled receptors. Methods in Neuroscience.

[25] Hummer G, Szabo A (2015). Optimal Dimensionality Reduction of Multistate Kinetic and Markov-State Models. The Journal of Physical Chemistry B.

[26] Hardt SL (1979). Rates of diffusion controlled reactions in one, two and three dimensions. Biophys Chem.

[27] Shoup D, Szabo A (1982). Role of diffusion in ligand binding to macromolecules and cell-bound receptors. Biophys J.

[28] Northrup SH, Allison SA, McCammon A (1984). Brownian dynamics simulation of diffusion‐influenced bimolecular reactions. The Journal of Chemical Physics.

[29] Spence, M. T. & Johnson I. D. The molecular probes handbook: a guide to fluorescent probes and labeling technologies. *Live Technologies Corporation* 38–39 (2010).

[30] Tabor A (2016). Visualization and ligand-induced modulation of dopamine receptor dimerization at the single molecule level. Sci Rep.

[31] Huang P (2001). Functional role of a conserved motif in TM6 of the rat mu opioid receptor: constitutively active and inactive receptors result from substitutions of Thr6.34(279) with Lys and Asp. Biochemistry.

[32] Kuszak AJ (2009). Purification and functional reconstitution of monomeric mu-opioid receptors: allosteric modulation of agonist binding by Gi2. J Biol Chem.

[33] Ko MC (2003). Studies of mu-, kappa-, and delta-opioid receptor density and G protein activation in the cortex and thalamus of monkeys. J Pharmacol Exp Ther.

[34] Wolfe LS, Morgan IG, Gombos G (1971). Isolation of plasma membranes from rat brain. Biochim Biophys Acta.

[35] Massotte D (2003). G protein-coupled receptor overexpression with the baculovirus-insect cell system: a tool for structural and functional studies. Biochim Biophys Acta.

[36] Massotte D (1997). Characterization of delta, kappa, and mu human opioid receptors overexpressed in baculovirus-infected insect cells. J Biol Chem.

[37] Stanasila L, Pattus F, Massotte D (1998). Heterologous expression of G-protein-coupled receptors: human opioid receptors under scrutiny. Biochimie.

[38] Manglik A (2012). Crystal structure of the micro-opioid receptor bound to a morphinan antagonist. Nature.

[39] Wu H (2012). Structure of the human kappa-opioid receptor in complex with JDTic. Nature.

[40] Wu B (2010). Structures of the CXCR4 chemokine GPCR with small-molecule and cyclic peptide antagonists. Science.

[41] Tan Q (2013). Structure of the CCR5 chemokine receptor-HIV entry inhibitor maraviroc complex. Science.

[42] Huang W (2015). Structural insights into mu-opioid receptor activation. Nature.

[43] Fiser A, Do RKG, Sali A (2000). Modeling of loops in protein structures. Protein Science.

[44] Fenalti G (2014). Molecular control of delta-opioid receptor signalling. Nature.

[45] Periole X, Cavalli M, Marrink SJ, Ceruso MA (2009). Combining an Elastic Network With a Coarse-Grained Molecular Force Field: Structure, Dynamics, and Intermolecular Recognition. Journal of Chemical Theory and Computation.

[46] Provasi D, Johnston JM, Filizola M (2010). Lessons from Free Energy Simulations of delta-Opioid Receptor Homodimers Involving the Fourth Transmembrane Helix. Biochemistry.

[47] Wassenaar TA, Ingólfsson HI, Böckmann RA, Tieleman DP, Marrink S (2015). J. Computational Lipidomics with insane: A Versatile Tool for Generating Custom Membranes for Molecular Simulations. Journal of Chemical Theory and Computation.

[48] Abraham MJ (2015). GROMACS: High performance molecular simulations through multi-level parallelism from laptops to supercomputers. SoftwareX.

[49] Bussi, G., Donadio, D. & Parrinello, M. Canonical sampling through velocity rescaling. *The Journal of Chemical Physics* **126**, (2007). 10.1063/1.2408420 17212484

[50] Berendsen HJC (1984). Molecular dynamics with coupling to an external bath. The Journal of Chemical Physics.

[51] Scherer MK (2015). PyEMMA 2: A Software Package for Estimation, Validation, and Analysis of Markov Models. Journal of Chemical Theory and Computation.

[52] Tribello, G. A., Bonomi, M., Branduardi, D., Camilloni, C. & Bussi, G. PLUMED2: New feathers for an old bird. *Comp. Phys. Comm*. **185** (2014).

[53] Goose JE, Sansom MS (2013). Reduced lateral mobility of lipids and proteins in crowded membranes. PLoS Comput Biol.

