# Supplementary material for: Molecular details of dimerization kinetics reveal negligible populations of transient µ-opioid receptor homodimers at physiological concentrations
Source: Sci Rep. 2018 May 16;8:7705. doi: 10.1038/s41598-018-26070-8 (PMC5955887; doi:10.1038/s41598-018-26070-8)
Supplement: Supplementary file 1 — Supplementary Information [file 41598_2018_26070_MOESM1_ESM.pdf]

SUPPLEMENTARY INFORMATION

Molecular details of dimerization kinetics reveal negligible populations of transient μ-opioid receptor homodimers at physiological concentrations

Derya Meral,[1] Davide Provasi,[1] Diego Prada-Gracia,[1] Jan Möller,[2] Kristen Marino,[1] Martin J. Lohse,[2,3] and Marta Filizola[1,*]

[1]*Department of Pharmacological Sciences, Icahn School of Medicine at Mount Sinai, New York, NY, USA.*

[2]*Max Delbrück Center for Molecular Medicine, Berlin, Germany.*

[3]*Institute of Pharmacology and Toxicology, Würzburg, Germany.*

Supplementary Figure 1	pg. 2
Supplementary Figure 2	pg. 3
Supplementary Figure 3	pg. 4
Supplementary Figure 4	pg. 5
Supplementary Figure 5	pg. 6
Supplementary Figure 6	pg. 7
Supplementary Figure 7	pg. 8
Supplementary Figure 8	pg. 9
Supplementary Figure 9	pg. 10
Supplementary Figure 10	pg. 11
Supplementary Figure 11	pg. 12
Supplementary Figure 12	pg. 13
Supplementary Table 1	pg.14
Supplementary Table 2	pg.15
Supplementary Table 3	pg.16
Supplementary Table 4	pg.17
Supplementary Table 5	pg.18
Supplementary Table 6	pg.19

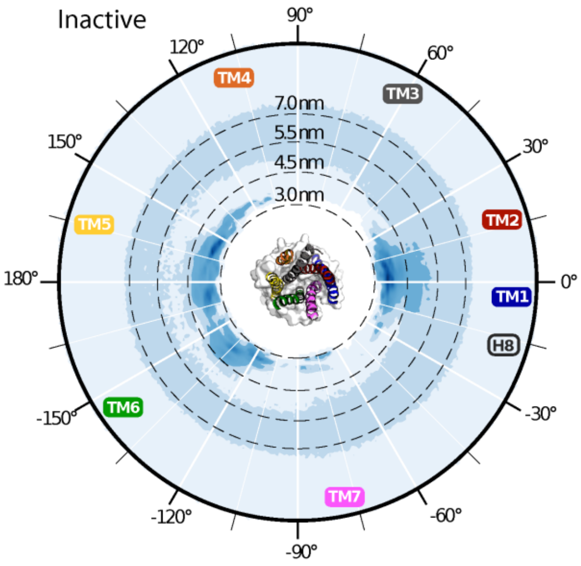

Supplementary Figure 1. Distributions of the CVs as monitored during the unbiased simulations of inactive and active MOR. Specifically, the probability to observe a second protomer at a given x,y position is calculated as: $1/(2N) \sum_{i,t} \delta(x - d(t)\cos\alpha_i(t))\ \delta(y - d(t)\sin\alpha_i(t))$, where N is the total number of frames t of the unbiased simulations and i=1,2 is the index of the protomer.

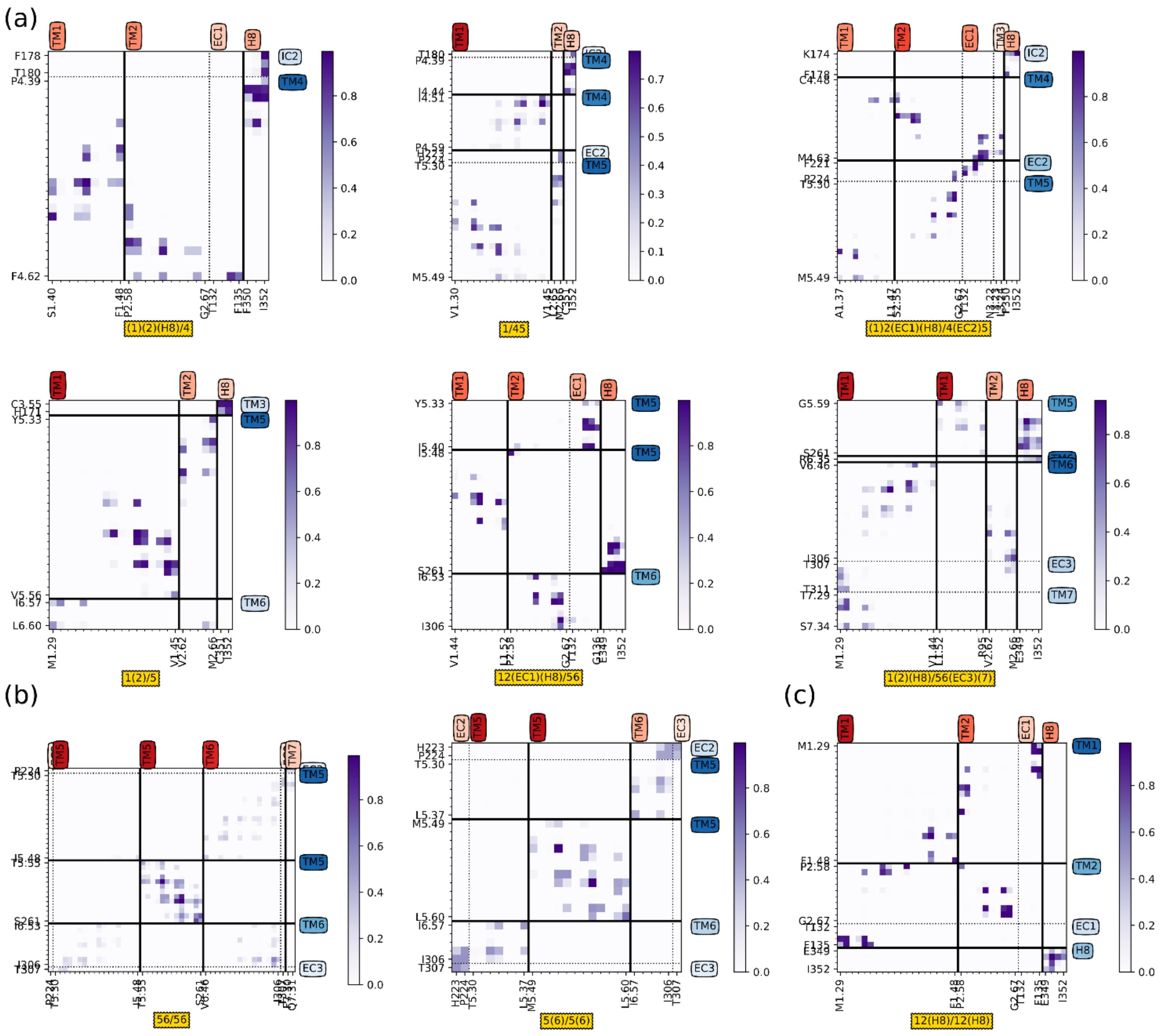

Supplementary Figure 2. Contact maps of the dimeric macrostates of MOR dimers formed by inactive protomers.

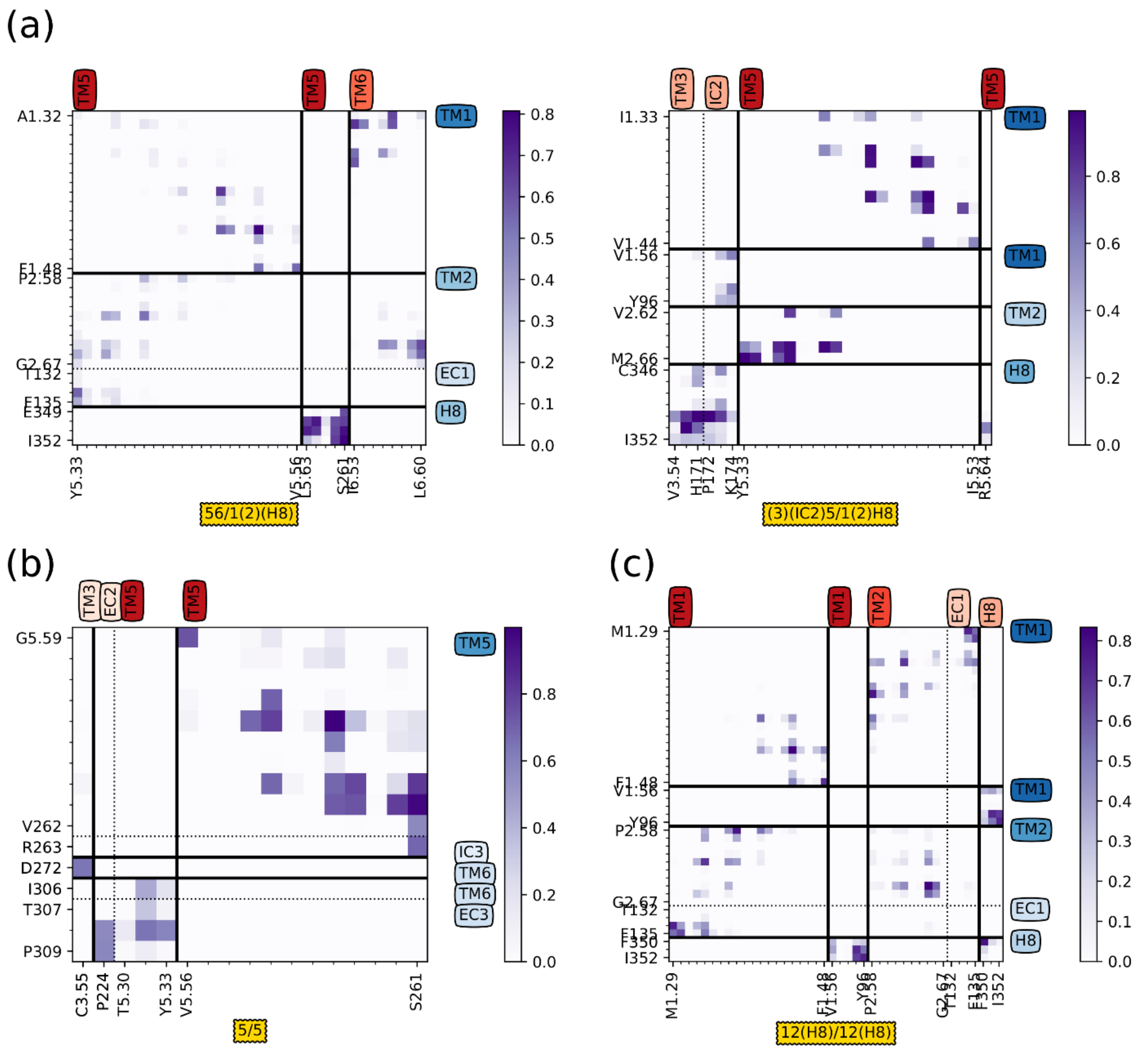

Supplementary Figure 3. Contact maps of the dimeric macrostates of MOR dimers formed by activated protomers.

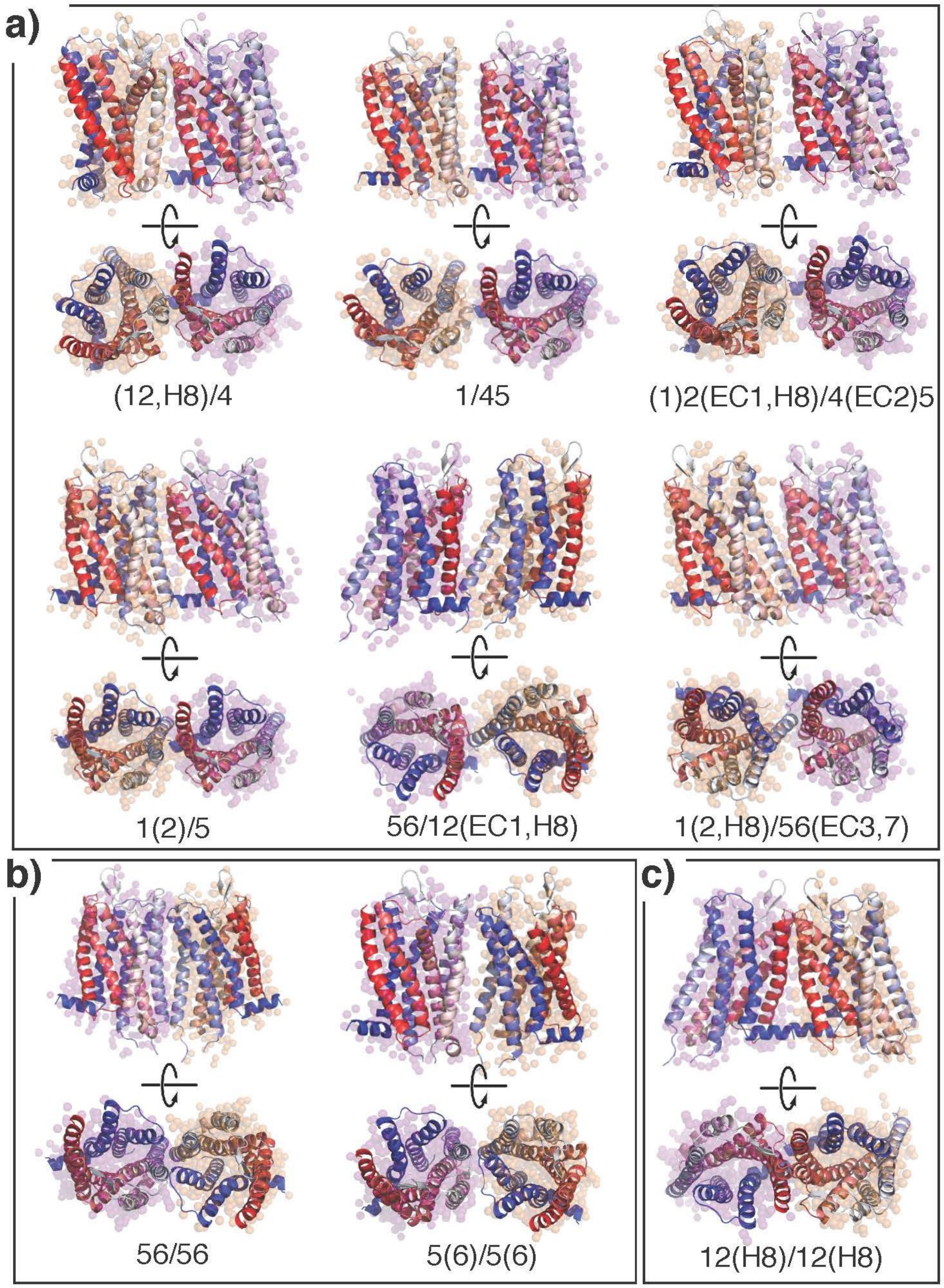

Supplementary Fig 4. Representative structures of the dimeric macrostates of the inactive MOR system. The cartoon representation of the inactive crystal structure (PDB ID: 4DKL), colored from red (TM1) to blue (H8), is aligned to the backbone beads of the coarse-grained structure by minimizing the RMSD between C_α atoms. Side-chain beads of the CG structure are represented as transparent spheres, whereas the backbone beads of the CG structure are not displayed for clarity.

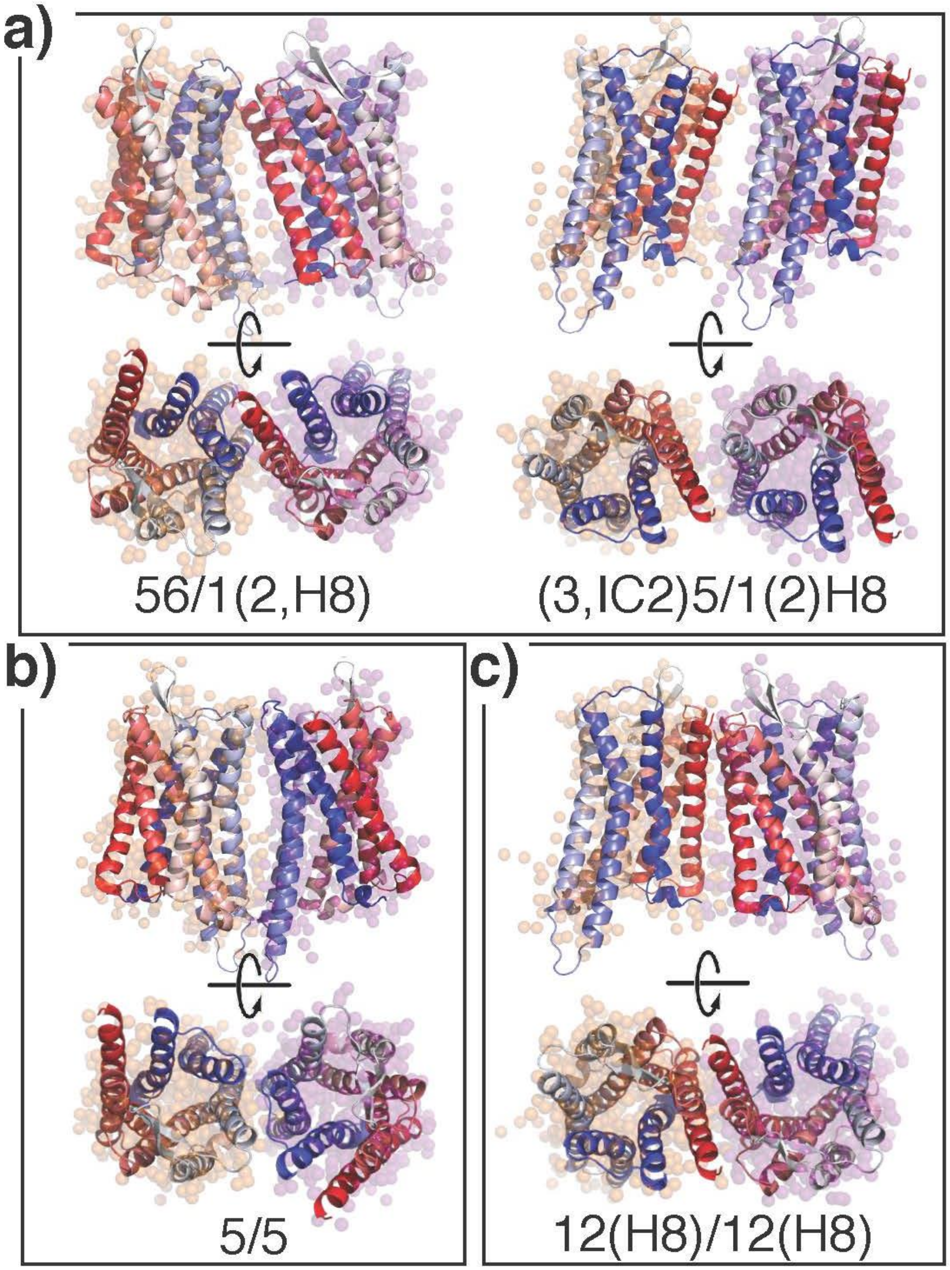

Supplementary Fig 5. Representative structures of the dimeric macrostates of the active MOR system. The cartoon representation of the active crystal structure (PDB ID: 5C1M), colored from red (TM1) to blue (H8), is aligned to the backbone beads of the coarse-grained structure by minimizing the RMSD between C_α atoms. Side-chain beads of the CG structure are represented as transparent spheres, whereas the backbone beads of the CG structure are not displayed for clarity.

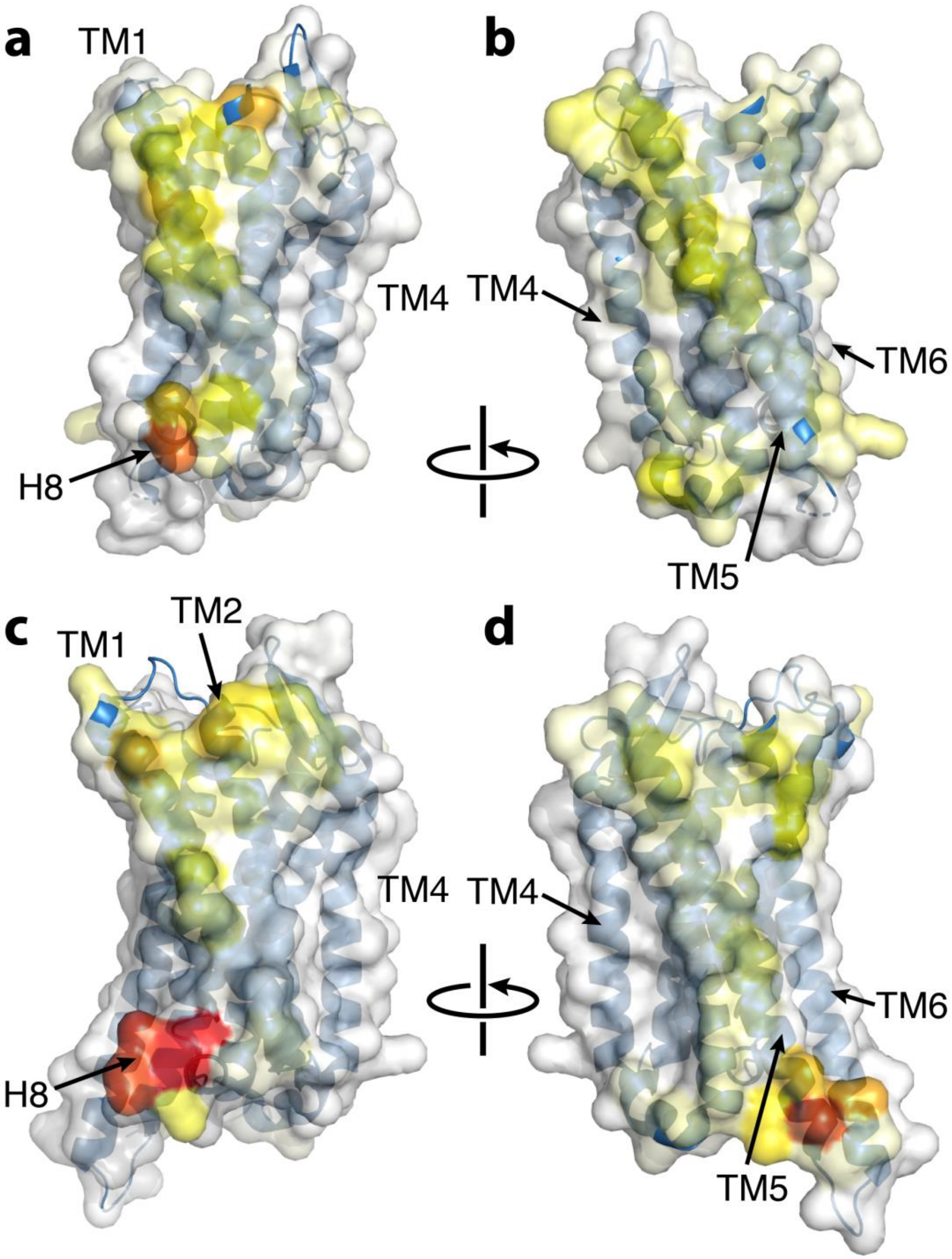

Supplementary Figure 6. Residues represented according to the average number of inter-protomer contacts formed at the interface of putative (a, b) inactive and (c, d) active MOR dimers calculated over all microstates (see Supplementary Table 2), where red represents the highest possible average contact number (1.76) and white represents zero.

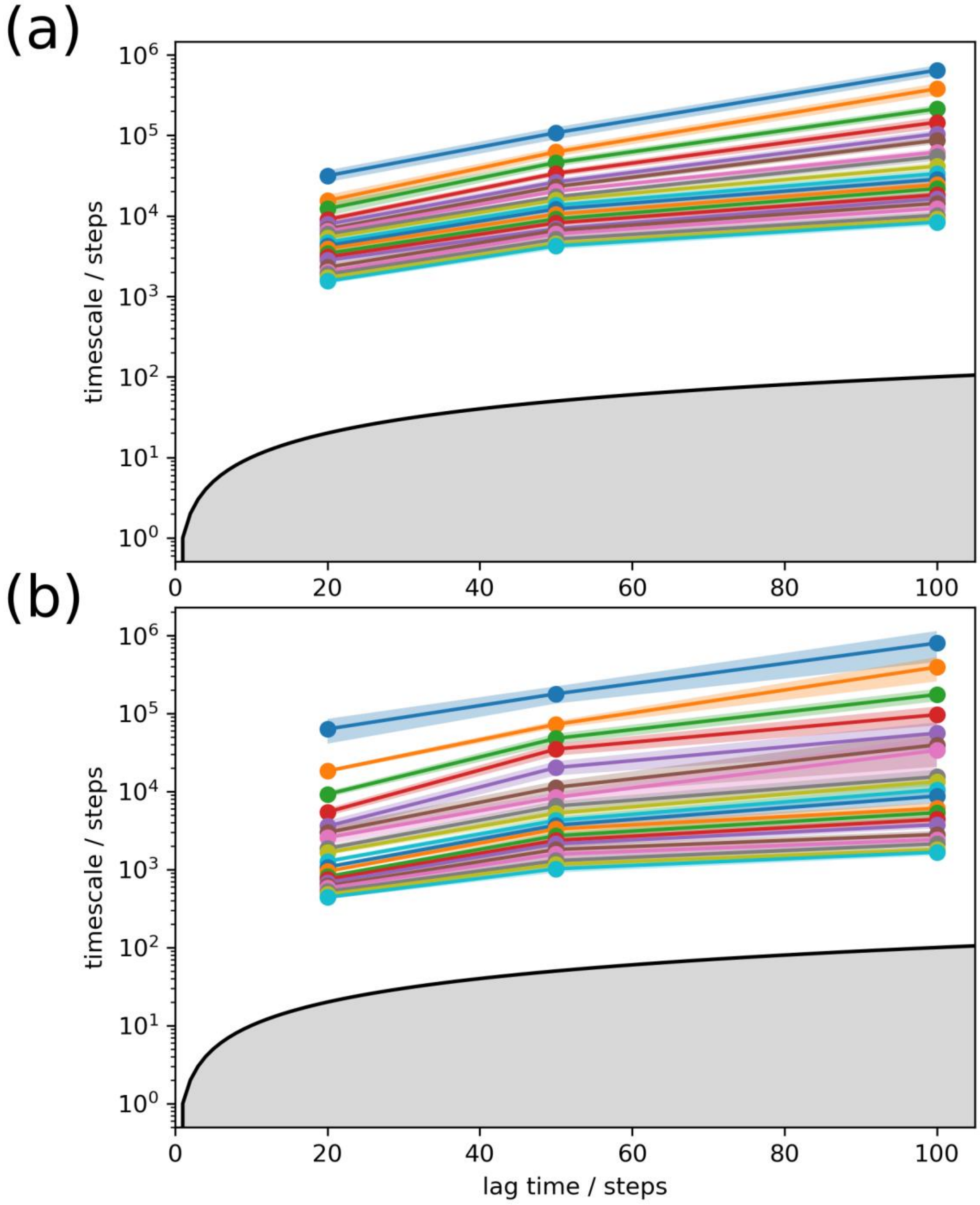

Supplementary Figure 7. TRAM convergence of the implied timescales with errors calculated through bootstrapping for the (a) inactive and (b) activated MOR structures.

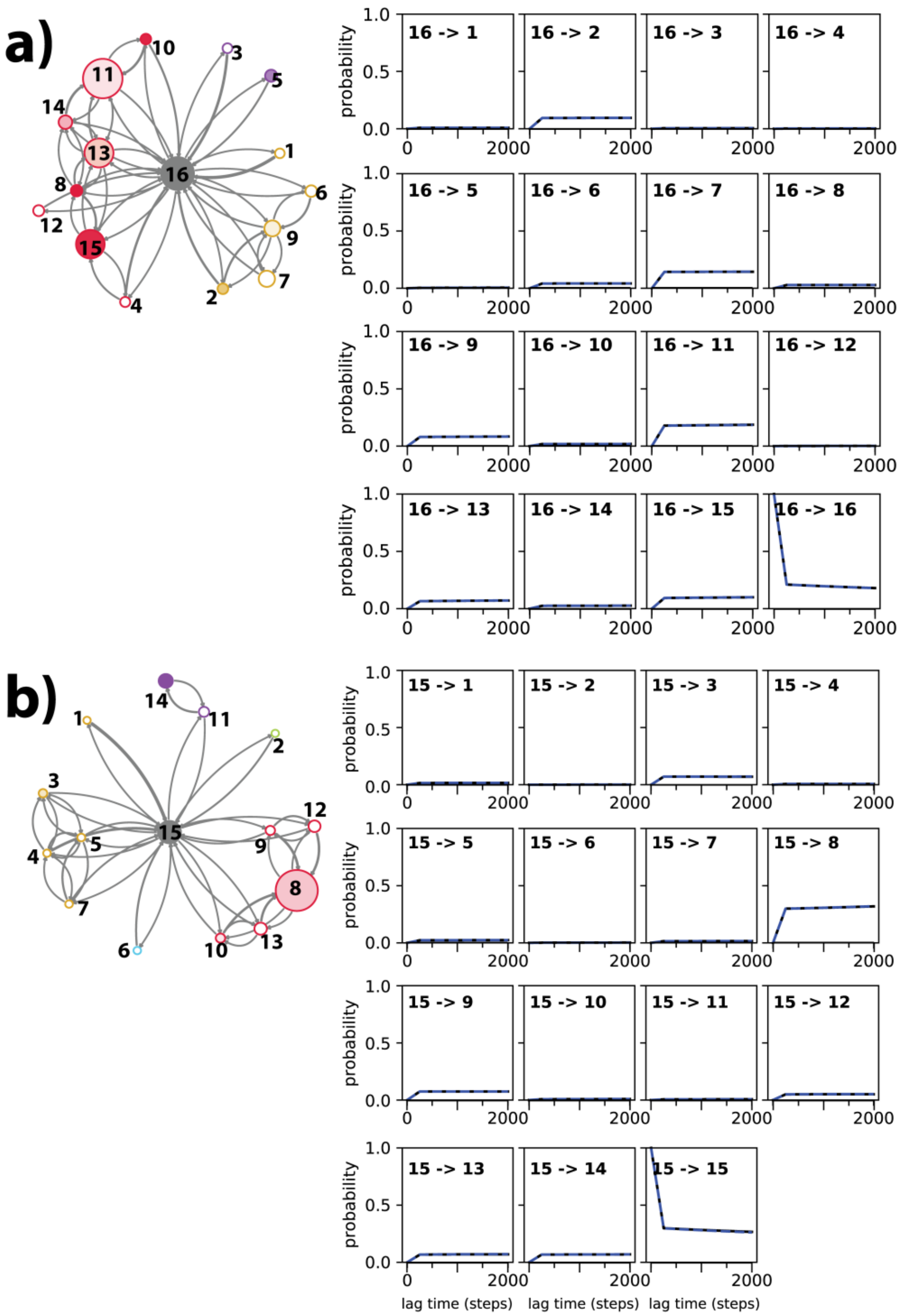

Supplementary Figure 8. Chapmann-Kolmogorov test applied to the TRAM kinetic model for transitions from the unbound state to each macrostate of the (a) inactive and (b) activated MOR derived from simulations.

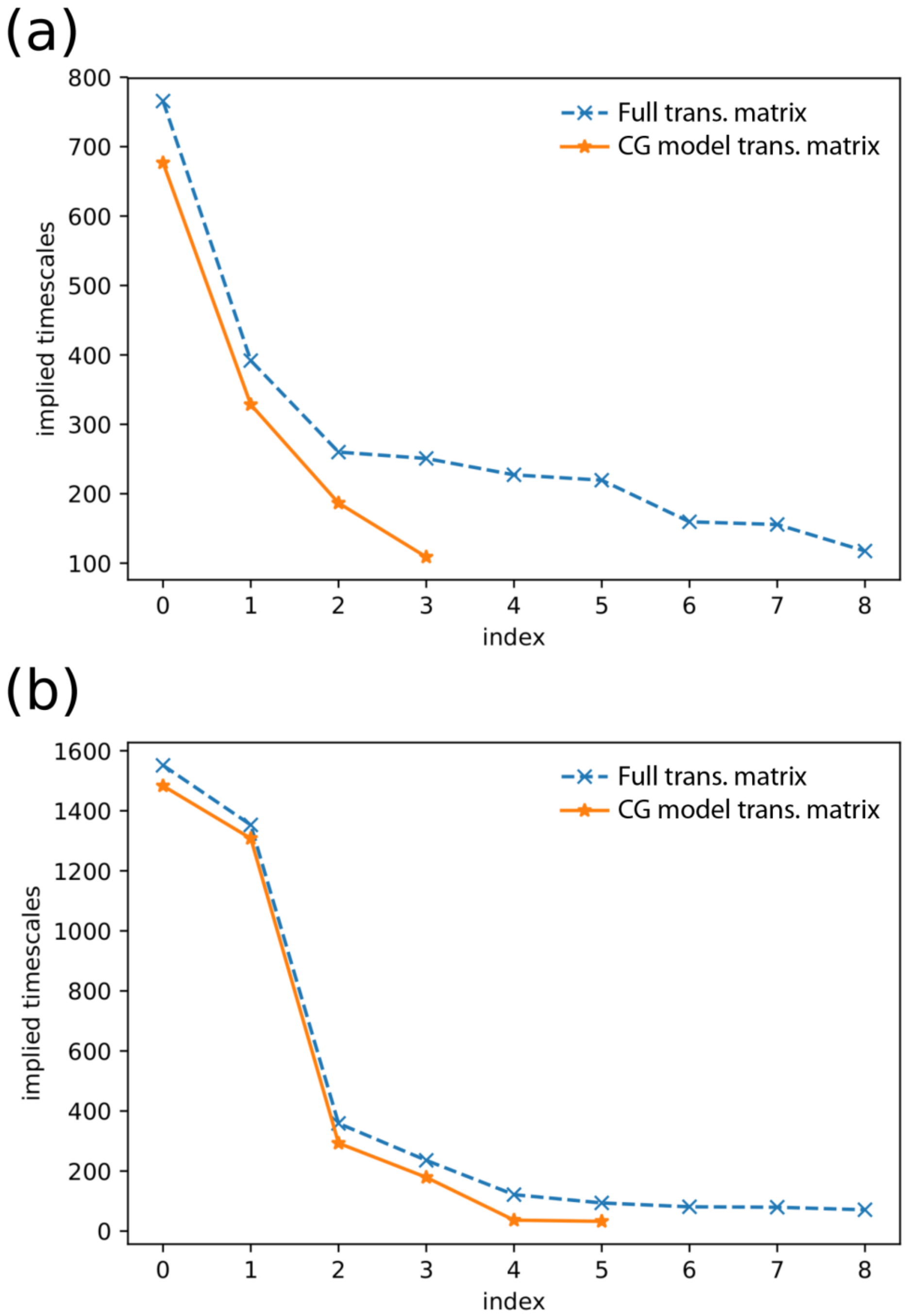

Supplementary Figure 9. Implied timescales of the coarse-grained model as compared to the timescales of the full transition matrix of the TRAM analysis for the (a) inactive and (b) activated MOR structures.

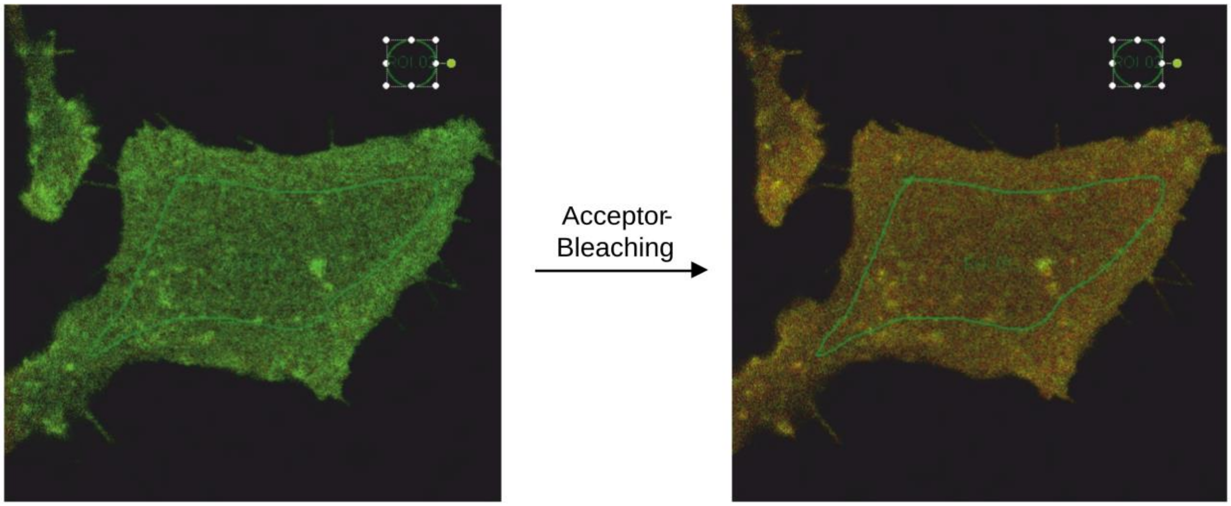

SNAP-CD28	ROI.01	ROI.02
Donor Pre	44.02	0.58
Donor Post	56.01	0.54
Acceptor Pre	111.3	0.94
Acceptor Post	46.32	0.88
Efficiency	0.2141	0

$$\textit{FRET efficiency} = \frac{I_{Donor\ (postbleach)} - I_{Donor\ (prebleach)}}{I_{Donor\ (postbleach)}}$$

Supplementary Figure 10. FRET acceptor photobleaching in confocal microscopy using SNAP-Surface® 549 as donor and SNAP-Surface® Alexa Fluor® 647 as acceptor. FRET-efficiencies are calculated according to the formula in the figure. Shown as example is a bleaching experiment with CD28.

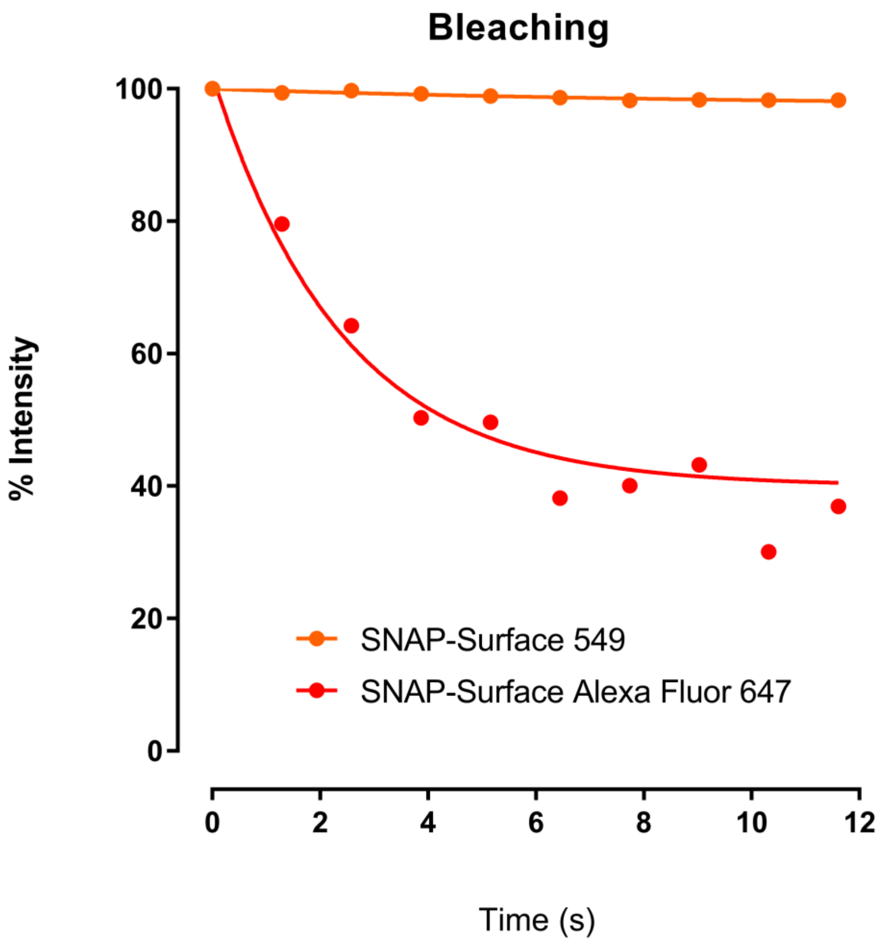

Supplementary Figure 11. Intensities of isolated Snap-dyes during bleaching in FRET-AB experiments. During bleaching the acceptor loses over 60% of intensity whereas the donor stays sovereignly stable (bleaching less than 2%).

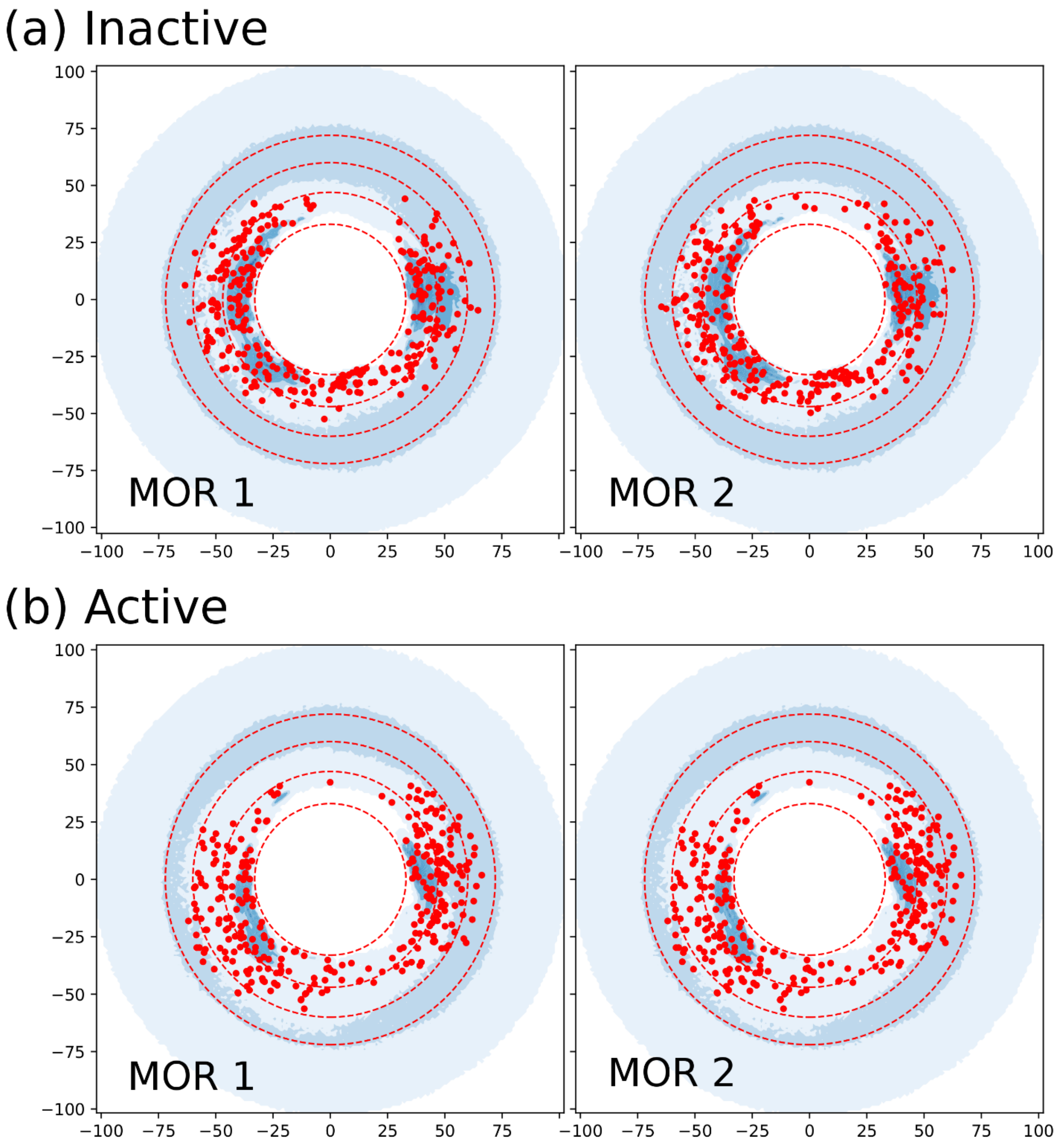

Supplementary Figure 12. Location of the umbrella centers selected for further simulations of the (a) inactive and (b) active MOR structures.

Supplementary Table 1. Summary of simulations.

		**Inactive MOR**			**Active MOR**		
		Count	***Length***	***Bias (d, α)***	***Count***	***Length***	***Bias (d, α)***
**Unbiased Simulations**		352	5 μs	-	352	5 μs	-
	Total	1.76 ms			1.76 ms		
**Biased Simulations**		68	0.3 μs	250kJ/mol/nm^2, 100kJ/mol	54	0.3 μs	250kJ/mol/nm^2, 100kJ/mol
		255	0.3 μs	100kJ/mol/nm^2, 80kJ/mol	260	0.3 μs	100kJ/mol/nm^2, 80kJ/mol
	Total	96.9 μs			94.2 μs		
Grand Total		1.86 ms			1.85 ms		

Supplementary Table 2. Residues at the interface of putative inactive or active MOR dimers that are involved in the largest average number (> 0.20) of inter-protomer contacts calculated over all microstates (see Methods for details). The residues common to both conformations have been marked in red.

Inactive MOR			Active MOR		
Residue	Position*	Average Number of Contacts	Residue	TM	Average Number of Contacts
I352	H8	1.302	F350	H8	1.757
M130	2.66	0.925	C351	H8	1.536
C351	H8	0.823	I352	H8	1.475
A73	1.37	0.815	S261	IL3	1.412
I238	5.44	0.663	K260	IL3	0.994
L129	2.65	0.651	I69	1.33	0.859
S76	1.40	0.643	L257	5.63	0.845
K174	IL2	0.595	L129	2.65	0.798
M243	5.49	0.595	R258	5.64	0.688
F350	H8	0.549	M130	2.66	0.675
I69	1.33	0.544	I298	6.53	0.610
Y227	5.33	0.513	I302	6.57	0.588
L231	5.37	0.431	L305	6.60	0.481
H223	EL2	0.416	V80	1.44	0.464
V80	1.44	0.406	E349	H8	0.443
I77	1.41	0.400	M72	1.36	0.372
C235	5.41	0.351	Y227	5.33	0.371
L246	5.52	0.349	A68	1.32	0.371
H171	IL2	0.324	P134	EL1	0.355
M72	1.36	0.317	L246	5.52	0.330
V126	2.62	0.301	V126	2.62	0.326
K260	IL3	0.294	M65	1.29	0.293
I242	5.48	0.292	A73	1.37	0.260
P224	EL2	0.278	F84	1.48	0.252
W228	5.34	0.273	F135	EL1	0.228
L257	5.63	0.272	S76	1.40	0.227
P134	EL1	0.268	P122	2.58	0.216
I234	5.40	0.266	Y299	6.54	0.209
V66	1.30	0.231	I301	6.56	0.201
F239	5.45	0.214			
M65	1.29	0.207			
I256	5.62	0.203			

*Residue position in TMs follows the Ballesteros-Weinstein generic numbering scheme.

Supplementary Table 3. Likelihood (>0.10) of inter-protomer contacts formed between inactive or active MOR protomers calculated over all microstates (see Methods for details).

Inactive MOR		Active MOR	
Contact	Probability	Contact	Probability
K174(IL2)-I352(H8)	0.253	S261(IL3)-I352(H8)	0.383
M130(2.66)-Y227(5.33)	0.234	S261(IL3)-F350(H8)	0.357
A73(1.37)-I238(5.44)	0.234	L257(5.63)-C351(H8)	0.356
L129(2.65)-Y227(5.33)	0.167	K260(IL3)-I352(H8)	0.346
H171(IL2)-I352(H8)	0.166	S261(IL3)-C351(H8)	0.343
M130(2.66)-P224(EL2)	0.163	R258(5.64)-F350(H8)	0.336
L129(2.65)-L231(5.37)	0.157	L257(5.63)-F350(H8)	0.330
K174(IL2)-C351(H8)	0.148	S261(IL3)-E349(H8)	0.319
I77(1.41)-M243(5.49)	0.142	K260(IL3)-C351(H8)	0.314
V173(IL2)-I352(H8)	0.140	K260(IL3)-F350(H8)	0.305
S76(1.40)-M243(5.49)	0.138	R258(5.64)-C351(H8)	0.250
M130(2.66)-W228(5.34)	0.125	I69(1.33)-I298(6.53)	0.217
A73(1.37)-M243(5.49)	0.123	M130(2.66)-L305(6.60)	0.189
H171(IL2)-C351(H8)	0.123	L129(2.65)-L305(6.60)	0.188
M130(2.66)-H223(EL2)	0.122	A68(1.32)-I302(6.57)	0.184
S76(1.40)-I238(5.44)	0.119	I69(1.33)-I302(6.57)	0.165
I69(1.33)-I234(5.40)	0.116	I69(1.33)-Y299(6.54)	0.160
A73(1.37)-F239(5.45)	0.102	M72(1.36)-I298(6.53)	0.159
		A73(1.37)-I298(6.53)	0.157
		V80(1.44)-L246(5.52)	0.153
		L257(5.63)-I352(H8)	0.149
		V262(IL3)-I352(H8)	0.138
		L129(2.65)-I302(6.57)	0.135
		R263(IL3)-I352(H8)	0.115

Supplementary Table 4. Decomposition of the binding pathways for the inactive MOR system. State numbers refer to the indexes in Supplementary Figure 8a. The fraction of flux corresponding to the direct transition from the unbound state is highlighted in bold.

**Cmp**	**Final state**	**Path(s)**
C_{00}	12(8)/12(8) (#5)	[16,5] (**94%**); [16,3,5] (5%)
$C_{\pi\pi}$	56/56 (#2)	[16,2] (**64%**); [16,9,2] (25%); [16,7,9,2] (7%)
	5(6)/5(6) (#9)	[16,9] (**61%**); [16,7,9] (18%); [16,2,9] (13%); [16,6,9] (7%)
$C_{0\pi}$	1(2,H8)/56(EC3,7) (#15)	[16,15] (**58%**); [16,13,15] (22%); [16,11,13,15] (12%); [16,11,13,8,15] (2%); [16,8,15] (2%)
	12(EC1,H8)/56 (#8)	[16,15,8] (41%); [16,13,8] (27%); [16,11,13,15,8] (13%); [16,8] (**8%**); [16,11,13,8] (5%)
	1(2)/5 (#13)	[16,13] (**43%**); [16,11,13] (29%); [16,15,13] (19%); [16,10,11,13] (3%); [16,14,11,13] (2%)
	(1)2(EC1,H8)/4(EC2)5 (#14)	[16,11,14] (37%); [16,13,11,14] (34%); [16,15,13,11,14] (14%); [16,14] (**7%**); [16,10,11,14] (3%)
	(128)/45 (#10)	[16,10] (**36%**); [16,11,10] (26%); [16,13,11,10] (24%); [16,15,13,11,10] (10%)
	1/45 (#11)	[16,11] (39%); [16,13,11] (36%); [16,15,13,11] (15%); [16,10,11] (4%); [16,14,11] (3%)

Supplementary Table 5. Decomposition of the binding pathways for the active MOR system. State numbers refer to the indexes in Supplementary Figure 8b. The fraction of flux corresponding to the direct transition from the unbound state is highlighted in bold.

**Cmp**	**Final state**	**Path(s)**
C_{00}	12(8)/12(8) (#14)	[15,11,14] (99%)
$C_{\pi\pi}$	5/5 (#3)	[15,4,3] (37%); [15,5,3] (25%); [15,3] (**23%**); [15,4,5,3] (7%); [15,7,5,3] (6%)
$C_{0\pi}$	(3,IC2)5/1(2) (#10)	[15,12,8,10] (35%); [15,13,10] (28%); [15,13,8,10] (15%); [15,10] (**10%**); [15,9,8,10] (9%)
	56/1(28) (#8)	[15,13,8] (41%); [15,12,8] (38%); [15,9,8] (10%); [15,10,8] (7%)

Supplementary Table 6. Significance analysis of the FRET-efficiencies of SNAP-labeled MOR constructs.

Number of families	1				
Number of comparisons per family	15				
Alpha	0.05				
Tukey's multiple comparisons test	Mean Diff.	95.00% CI of diff.	Significant?	Summary	Adjusted P Value
CD28 vs. β1AR	0.1683	0.1451 to 0.1914	Yes	****	<0.0001
CD28 vs. MOR-wt	0.1731	0.1519 to 0.1943	Yes	****	<0.0001
CD28 vs. T279D	0.1698	0.1476 to 0.192	Yes	****	<0.0001
CD28 vs. T279K	0.1481	0.1254 to 0.1708	Yes	****	<0.0001
β1AR vs. MOR-wt	0.004833	-0.01792 to 0.02759	No	ns	0.9902
β1AR vs. T279D	0.001562	-0.0221 to 0.02523	No	ns	>0.9999
β1AR vs. T279K	-0.02013	-0.04428 to 0.004028	No	ns	0.1621
MOR-wt vs. T279D	-0.003272	-0.02509 to 0.01855	No	ns	0.9981
MOR-wt vs. T279K	-0.02496	-0.04731 to -0.002611	Yes	*	0.0188
T279D vs. T279K	-0.02169	-0.04497 to 0.001585	No	ns	0.0835
